# Histidine kinases mediate differentiation, stress response, and pathogenicity in *Magnaporthe oryzae*

**DOI:** 10.1002/mbo3.197

**Published:** 2014-08-08

**Authors:** Stefan Jacob, Andrew J Foster, Alexander Yemelin, Eckhard Thines

**Affiliations:** 1 Institute of Biotechnology and Drug Research (IBWF)Erwin-Schrödinger-Str. 56, D-67663, Kaiserslautern, Germany; 2 Johannes Gutenberg-University Mainz, Institute of Biotechnology and Drug ResearchDuesbergweg 10-14, D-55128, Mainz, Germany

**Keywords:** Appressoria, conidia, differentiation, histidine kinase, HOG pathway, hypoxia signaling, *Magnaporthe oryzae*, pathogenicity

## Abstract

The aim of this study is a functional characterization of 10 putative histidine kinases (HIKs)-encoding genes in the phytopathogenic fungus *Magnaporthe oryzae*. Two HIKs were found to be required for pathogenicity in the fungus. It was found that the mutant strains *ΔMohik5* and *ΔMohik8* show abnormal conidial morphology and furthermore *ΔMohik5* is unable to form appressoria. Both HIKs MoHik5p and MoHik8p appear to be essential for pathogenicity since the mutants fail to infect rice plants. MoSln1p and MoHik1p were previously reported to be components of the HOG pathway in *M. oryzae*. The *ΔMosln1* mutant is more susceptible to salt stress compared to *ΔMohik1*, whereas *ΔMohik1* appears to be stronger affected by osmotic or sugar stress. In contrast to yeast, the HOG signaling cascade in phytopathogenic fungi apparently comprises more elements. Furthermore, vegetative growth of the mutants *ΔMohik5* and *ΔMohik9* was found to be sensitive to hypoxia-inducing NaNO_2_-treatment. Additionally, it was monitored that NaNO_2_-treatment resulted in MoHog1p phosphorylation. As a consequence we assume a first simplified model for hypoxia signaling in *M. oryzae* including the HOG pathway and the HIKs MoHik5p and MoHik9p.

## Introduction

**Figure 1:**
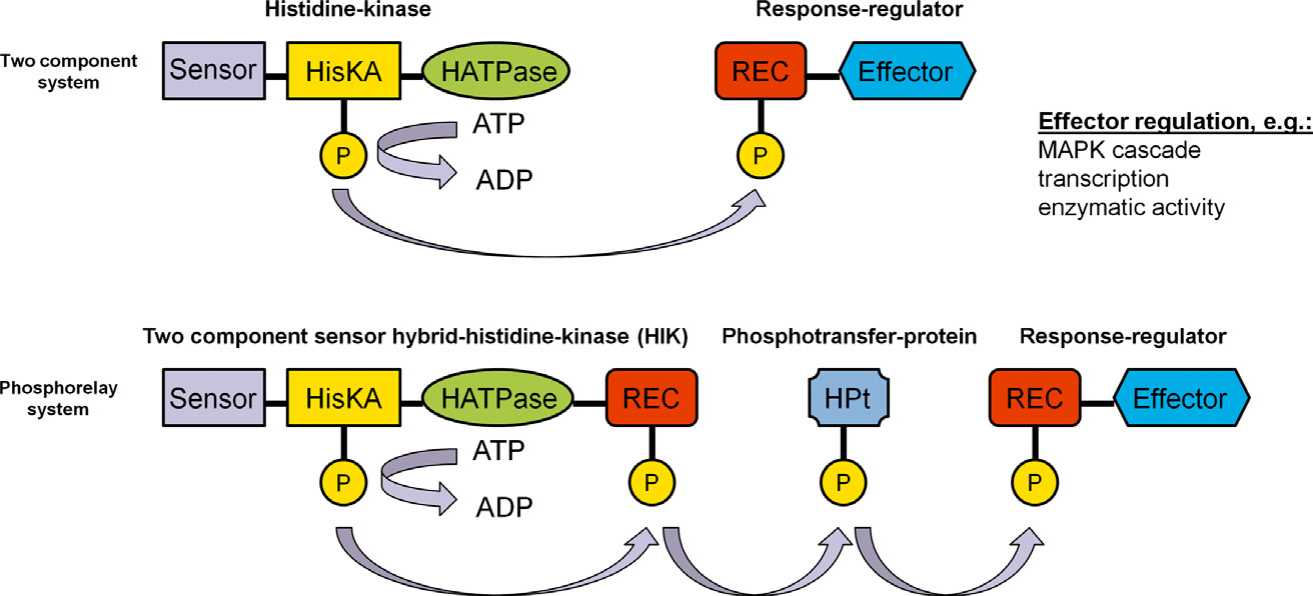
Modular scheme of the basic two component system (TCS) and the phosphorelay system (PS). The sensor domain (sensor), the histidine kinase phosphoacceptor domain (HisKA), the histidine-like ATPase domain (HATPase), the signal receiver domain (REC), the histidine-containing phosphotransfer domain (HPt), and the effector domain (effector) are shown in different colors. The phosphoryl group transfer is indicated by arrows. The PS includes additional regulatory steps, the phosphoryl group is transferred from the HisKA to a REC domain of the hybrid histidine kinase and subsequently transferred via a phosphotransfer protein to the response regulator (modified from Catlett et al. 2003).

Detection of external stimuli and the transfer of these signals within the cell to give an appropriate response are vital for organisms in order to adapt to varying environmental conditions during their life cycles. The essence of signal transduction is processing of signal detection to gene activation or other cellular responses (Hoch 2000). In order to detect and transform external signals into transducible cellular events, organisms developed sensor proteins, for example, two component sensor hybrid histidine kinases (HIKs). Signaling mechanisms are found in all living cells as two component systems or as phosphorelay systems in more complex organisms (Fig.1). Such systems confere signal transfer of a phosphoryl group from an, often membrane bound, histidine kinase to a response regulator protein and thus trigger various physiological responses. Phosphorylation can promote oligomerization (Weiss et al. 1992; Webber and Kadner 1997), dimerization (Cobb and Goldsmith 2000), interactions with other proteins (Blat and Eisenbach 1994; Newton 2001), interactions with DNA (Aiba et al. 1989), or combinations of these mechanisms (Harlocker et al. 1995; Anand et al. 1998). The physiological responses range from activation of mitogen acivated protein kinases (MAPK) and regulation of transcription factors up to modulation of enzymatic activity (Stock et al. 2000; Wolanin et al. 2002). Phosphorelay systems have been implicated in regulating differentiation processes, chemotaxis, secondary metabolite production, and virulence-associated processes in pathogenic and nonpathogenic bacteria and fungi (Grebe and Stock 1999; Wolanin et al. 2002). HIK- or response regulator-encoding genes have not been identified in the animal kingdom as a whole (Lander et al. 2001; Wolanin et al. 2002). In fungi, HIKs are classified into 11 groups (Catlett et al. 2003) and known as mediators of environmental stress responses, pathogenicity, hyphal development, and sporulation (Li et al. 1998; Hohmann 2002; Nemecek et al. 2006; Viaud et al. 2006; Islas-Flores et al. 2011). Furthermore, members of group III HIKs are believed to be the target of commercial pesticides such as fludioxonil, iprodione, and the antifungal natural product ambruticin (Yoshimi et al. 2004; Motoyama et al. 2005b; Dongo et al. 2009; Fillinger et al. 2012).

Even though sensor HIKs are related to Ser/Thr/Tyr protein kinases, they are structurally distinct and differ in their chemistry: Ser/Thr/Tyr kinases form phosphoesters, whereas HIKs produce phosphoramidate linkages (Islas-Flores et al. 2011). Thereby hydrolysis of phosphoramidates yields significantly higher negative free energy compared to the hydrolysis of phosphoesters, and the use of these higher energy potentials in biological systems differs accordingly (Stock et al. 1990). A *γ*-phosphoryl group of ATP is transferred to the imidazole ring of the histidine residue within a phosphoacceptor domain of the histidine kinase (HisKA) and the response-regulator protein catalyzes the transfer of this phosphoryl group to an aspartic acid residue within a signal receiver domain (REC) of the response regulator (Fig.1). As a consequence, the effector domain of the response regulator initiates the subsequent regulation. In many eukaryotic PS ancillary histidine-containing phosphotransfer domains (HPt) within a phosphotransfer protein act as interim stages prior to phosphoryl group transfer to the REC of the response regulator protein (Appleby et al. 1996) (Fig.1).

HIKs are supposed to originate from bacteria. Two component systems are found in almost all bacterial species and phylogenetic analysis implies that bacterium-to-eukaryote horizontal gene transfer had occurred between ancestors of these organisms (Brinkman et al. 2001). Many authors expect the number of two component genes to correlate strongly with ecological and environmental niches of the organisms (Koretke et al. 2000; Galperin 2005; Alm et al. 2006; Galperin et al. 2010). Bacteria living predominantly in constant environmental niches typically comprise only a limited number of two component system genes. Obligate intracellular pathogens or endosymbionts possess only a few genes encoding two component signaling systems or sometimes none at all (i.e., *Mycoplasma genitalium*) (Ulrich and Zhulin 2010). These findings indicate the evolutionary adaption of organisms to rapid changing environmental conditions by acquisition of their individual sets of two component systems. This signaling mechanism was until 1993 believed to be restricted to prokaryotes (Parkinson 1993), but thereafter phosphorelay systems comprising HIKs were identified in the plant *Arabidopsis thaliana* and in *Saccharomyces cerevisiae* (Chang et al. 1993; Chang and Meyerowitz 1994; Maeda et al. 1994; Urao et al. 2001).

The characterization of fungal two component signaling systems and the HIKs involved, especially in phytopathogenic fungi, has not been addressed to large extent. In the genome of *S. cerevisiae* only one HIK (Sln1p) has been identified, which is part of the phosphorelay system (Sln1p-Ypd1p-Ssk1p) in the high-osmolarity glycerol (HOG) pathway (Maeda et al. 1994; Posas et al. 1996; Hohmann 2002). Because HIKs are vital for coordination of distinct changes in their life cycle, facultative pathogenic microorganisms often have more HIK-encoding genes due to the frequently and rapidly environmental changes during host penetration and colonization. *Candida albicans* was found to contain three HIKs (Hk1p, Sln1p, Os1p/Nik1p) involved in osmoregulation, hyphal development, and virulence (Nagahashi et al. 1998; Catlett et al. 2003). Sequence analysis predicts 10 HIK-encoding genes in the genome sequence of the filamentous fungus *Magnaporthe oryzae* (*Pyricularia oryzae*; Catlett et al. 2003; Dean et al. 2005).

*Magnaporthe oryzae* is the causal agent of rice blast disease. Asexual conidia of *M. oryzae* infect rice plants under conditions of relatively high humidity via an infection structure called appressorium. Rice blast results in crop losses up to 30% of the global rice yield every year (Dean et al. 2012). However, the molecular and biochemical basis of the infection-related morphogenesis of *M. oryzae* on the plant surface has been studied intensively within the last decades (Gilbert and Dean 1996; Dean 1997; Talbot 2003; Wang et al. 2005) in planta growth and adaption has not been addressed extensively. Functional HIKs are required for plant infection or invasive growth in planta, since their function appears to be essential for the adaption to environmental changes, for example, oxygen saturation, osmotic pressure, temperature, nutrition status, and reactive oxygen species (Islas-Flores et al. 2011). Therefore, HIKs appear to be promising fungicide targets, since they are present in pathogenic organisms, whereas absent in mammals. In *M. oryzae*, HIKs have not been thoroughly studied so far. It was found that inactivation of the gene *MoSLN1* results in a mutant *ΔMosln1*, which is susceptible to various stresses, altered in cell wall integrity and in pathogenicity (Zhang et al. 2010), and the group III HIK MoHik1p is a previously described target of commercial fungicides (Motoyama et al. 2005b). Therefore, it appears of interest to elucidate and characterize the functions of the remaining HIKs in *M. oryzae* to obtain new insights into the molecular basis of plant–pathogen interaction.

## Experimental Procedures

### Strains, growth conditions, and oligonucleotides

All mutants described in this study were generated from *M. oryzae* 70-15 strain (wild type, WT) (Fungal Genetics Stock Centre, Kansas City, MO). The strain and all mutant strains were grown at 26°C on complete medium (CM, pH 6.5, 2% agar) CM contains per liter: 10 g glucose, 1 g yeast extract, 2 g peptone, 1 g casamino acid, 50 mL nitrate salt solution (containing per liter H_2_O: 120 g NaNO_3_, 10.4 g KCl, 30.4 g KH_2_PO_4_, 10.4 g MgSO_4_ × 7 H_2_O), and 1 mL of a trace element solution (containing per liter H_2_O: 22 g ZnSO_4_ × 7 H_2_O, 11 g H_3_BO_3_, 5 g MnCl_2_ × 4 H_2_O, 5 g FeSO_4_ × 7 H_2_O, 1.7 g CoCl_2_ × 6 H_2_O, 1.6 g CuSO_4_ × 5 H_2_O, 1.5 g Na_2_MoO_4_ × 2 H_2_O, 50 g Na_2_EDTA, pH 6.5 adjusted by 1 mol/L KOH) (adapted and modified from Talbot et al. 1993).

All oligonucleotides used in this study are listed in Table S1 and were obtained from Eurofins-MWG-Operon (Ebersberg, Germany). All chemicals used were obtained from Sigma-Aldrich (Munich, Germany) unless otherwise stated.

### Identification and sequence analysis of HIKs in *Magnaporthe oryzae*

Sequence analysis and comparison of the histidine kinase genes in the *M. oryzae* genome were conducted via the *Magnaporthe* comparative Database (*Magnaporthe* comparative Sequencing Project, Broad Institute of Harvard and MIT [http://www.broadinstitute.org/], annotation *M. oryzae* 70-15 [MG8]). In addition, the conserved protein domains of the sequences were verified using algorithms searching against the CDD database on NCBI (http://www.ncbi.nlm.nih.gov/Structure/cdd/wrpsb.cgi) or the Pfam 27.0 database (http://pfam.sanger.ac.uk/). The prediction of transmembrane helices was implemented via TMHMM Server v2.0 (http://www.cbs.dtu.dk/services/TMHMM-2.0/) and the “DAS” transmembrane prediction server (http://www.sbc.su.se/∼miklos/DAS/maindas.html) to validate the results.

Phylogenetic analysis was carried by using the program MEGA 5.2 (Tamura et al. 2011). Sequence alignments were performed using ClustalW (Larkin et al. 2007). “BLOSUM” was used as “cost matrix” with “gap open cost”: 10 and “gap extend cost”: 0.1. Phylograms were made using the “neighbor-joining”-algorithm with the “Jones–Taylor–Thornton (JTT)-model” (Saitou and Nei 1987; Jones et al. 1992). The Bootstrapping analysis involved 1000 replicates. In order to visualize the data, “Geneious 6.1.7”-software (Biomatters, Auckland, New Zealand) was applied.

### DNA manipulation/construction of gene inactivation vectors

Genomic DNA was isolated from lyophilized mycelium of 4-day-old liquid cultures, grown at 26°C and 120 rpm, using DNeasy® Plant Mini Kit (Qiagen, Hilden, Germany) according to the manufacturer's instructions. DNA manipulation procedures were followed up by standard procedures (Green and Sambrook 2012). *Escherichia coli* XL1-BLUE strain (Stratagene Products Division, La Jolla, CA) was used for routine bacterial transformations and construction of plasmids.

Transformations of *M. oryzae* were conducted using *Agrobacterium tumefaciens*-mediated transformation (ATMT; De Groot et al. 1998; Rho et al. 2001). The detailed procedures followed those described previously (Odenbach et al. 2007). *Magnaporthe oryzae*-mutant strains were generated using a hygromycin resistance (hygromycin-phospho-transferase gene, *HPT*; Odenbach et al. 2007) or a glufosinate-ammonium resistance (phosphinothricin-acetyl-transferase gene, *BAR*; Kramer et al. 2009). For detailed information on the inactivation strategies as well as confirmation of successful gene replacement, interruption, see Data S1 and Figure S1, respectively.

### Vegetative growth assays/sensitivity assays

Agar blocks of 0.5-cm diameter were cut from the outer growth zone of the cultures to be tested and placed onto freshly prepared CM or minimal medium agar plates (MM, pH 6.5, 2% agar, contains per liter: 1 g glucose, 50 mL nitrate salt solution, 0.25 mL biotin solution [0.01%], 1 mL thiamindichloride solution [1%], and 1 mL of a trace element solution) with different stress inducing compounds. The cultures were grown for 10 days at 26°C and the colony diameter was measured.

### Appressorium formation assays and conidial morphology

Appressorium development was assayed by monitoring germination of conidia on hydrophobic plastic slides (thickness 1, 76 × 51 mm, no. 653081; Greiner Bio-One, Kremsmünster, Austria). For this purpose conidia were harvested from 11-day-old *M. oryzae* cultures grown on CM, filtered through two layers of miracloth to give a conidial suspension, which was adjusted to 5 × 10^4^ conidia/mL in H_2_O. Hundred microliter drops of this conidial suspension were placed on the plastic slides and incubated at room temperature. After 16 h the number of appressoria formed was counted. In addition, the same assay was carried out on glass slides upon chemical stimulation with 500 ng/mL 1,16-hexadecandiol (1,16-HDD, dissolved in MeOH). 1,16-HDD is a plant lipid or wax compound and an inducer of appressorium formation in *M. oryzae* (Gilbert and Dean 1996).

### Cell wall stability/protoplast assays

In order to assess cell wall stability *M. oryzae* cultures were grown in CM liquid medium for 3 days at 26°C and 120 rpm. Equal amounts of mycelium were washed in 20% sucrose solution. Three mg/mL lysing enzymes of *Trichoderma harzianum* (Sigma-Aldrich) were added followed up by incubation for 60 min at RT on a tilting shaker. After this incubation period, the samples were filtered through two layers of miracloth and centrifuged at 2900*g* for 10 min at 4°C. The resulting pellets were carefully resuspended in equal amounts of 20% sucrose solution and the number of protoplasts was counted.

### Plant infections/pathogenicity assays with rice

The plant infection assays were carried out using 21-day-old plants of dwarf indica rice cultivar CO-39. Plants were cultivated using a daily cycle of 16 h light followed up by 8 h darkness (28°C, 90% relative humidity). Conidial suspensions were adjusted to 5 × 10^4^ conidia/mL in H_2_O containing 0.2% gelatin. Five rice plants were spray inoculated with each 5 mL of conidial suspension and were incubated in plastic bags in a test chamber (Versatile Environmental Test Chamber MLR-350H; Sanyo Electric Co., Illinois, USA). After 5 days of incubation, lesions were counted. In planta growth was tested on wounded rice leaves. Therefore, conidial suspensions were added to wounded leaves and after 2 days, the leaves were photographed. Leaves were carefully wounded with fine sandpaper, granulation 240.

### Western blot analysis of phosphorylated MoHog1p

Phosphorylation of the MAPK MoHog1p in *M. oryzae* was analyzed by western blot analysis using an anti-Phospho-p38 MAPK (Thr180/Tyr182) (D3F9) XP™ Rabbit monoclonal antibody (Cell Signaling Technology, Beverly, MA). Five milliliter of CM liquid medium was inoculated with equal amounts of mycelium of *M. oryzae* strains in cell culture plates (6-well, Greiner Bio-One). After 65 h incubation at 26°C and 120 rpm the cultures were exposed to NaCl or sorbitol on a shaker for 10 min at RT. The cell suspensions were centrifuged at 2900 *g* for 10 min at 4°C. The supernatant was discarded and 300 *μ*L of SDS-loading dye (10 mmol/L Tris-HCl pH 6.8, 2.0% SDS, 5% glycerol, 0.1 mol/L dithiothreitol, 0.01% bromphenol blue) were added to the mycelium and heated to 100°C for 10 min. In order to break cell walls, glass beads were used in the Ribolyzer Fast Prep FP120 (Thermo Savant, Illkirch, France) for 30 sec at 6.0 Hz and a centrifugation step for 5 min at 11500 *g* followed. Equal amounts of the supernatant of the individual probe were separated by SDS-polyacylamide gel electrophoresis and blotted on a nitrocellulose transfer membrane (Roti®-NC; Carl Roth GmbH, Karlsruhe, Germany) using electrophoretic transfer (Mini Trans-Blot® Electrophoretic Transfer Cell; Bio-Rad Laboratories, Munich, Germany). Western immunoblotting was carried out with the Phototope®-HRP Western Blot Detection System (Cell Signaling Technology) according to the manufacturer's instructions.

## Results

The *M. oryzae*-mutant strains were generated by *Agrobacterium tumefaciens*-mediated transformation using the *HPT* gene (Odenbach et al. 2007) or the *BAR* gene (Kramer et al. 2009) to interrupt and to replace the HIK-encoding sequences (for detailed information see Experimental Procedures and the information in Data S1 and Fig. S1), respectively. An overview of all HIK-mutant strains used in this study with their most important phenotypes is given in Table1.

**Table 1:** Overview of the HIK mutants in *Magnaporthe oryzae* and their most important phenotypes.

Mutant name	MGG number of the HIK-encoding gene	Phenotypes/alterations in the mutant strain
*ΔMosln1*	MGG_07312	Osmosensitive (salt), conidiation, appressorium formation, cell wall integrity, pathogenicity, NaNO_2_ sensitive, ion stress (CuSO_4_), temperature sensitivity
*ΔMohik1*	MGG_11174	Osmosensitive (sugar), pathogenicity, fungicide resistance, NaNO_2_ sensitive, temperature sensitivity
*ΔMohik2*	MGG_01342	CoCl_2_ sensitive, ion stress (CuSO_4_), pathogenicity
*ΔMohik3*	MGG_12530	Pathogenicity
*ΔMohik4*	MGG_13891	CoCl_2_ sensitive, pathogenicity
*ΔMohik5*	MGG_11882	Conidia morphology, conidiation, cell wall integrity, apathogen, vegetative growth, CoCl_2_ and NaNO_2_ sensitive, ion stress (CuSO_4_)
*ΔMohik6*	MGG_06696	Pathogenicity
*ΔMohik7*	MGG_12377	None
*ΔMohik8*	MGG_01227	Conidia morphology, conidiation, apathogen, vegetative growth, CoCl_2_ sensitive
*ΔMohik9*	MGG_02665	Cell wall integrity, CoCl_2_ and NaNO_2_ sensitive
*ΔMohik11/sln1*	MGG_11174/MGG_07312	Osmosensitive (salt and sugar), conidiation, appressorium formation, cell wall integrity, pathogenicity, NaNO_2_ sensitive, ion stress (CuSO_4_), temperature sensitivity

HIK, histidine kinases.

### Identification and sequence analysis of HIKs in *M. oryzae*

**Figure 2:**
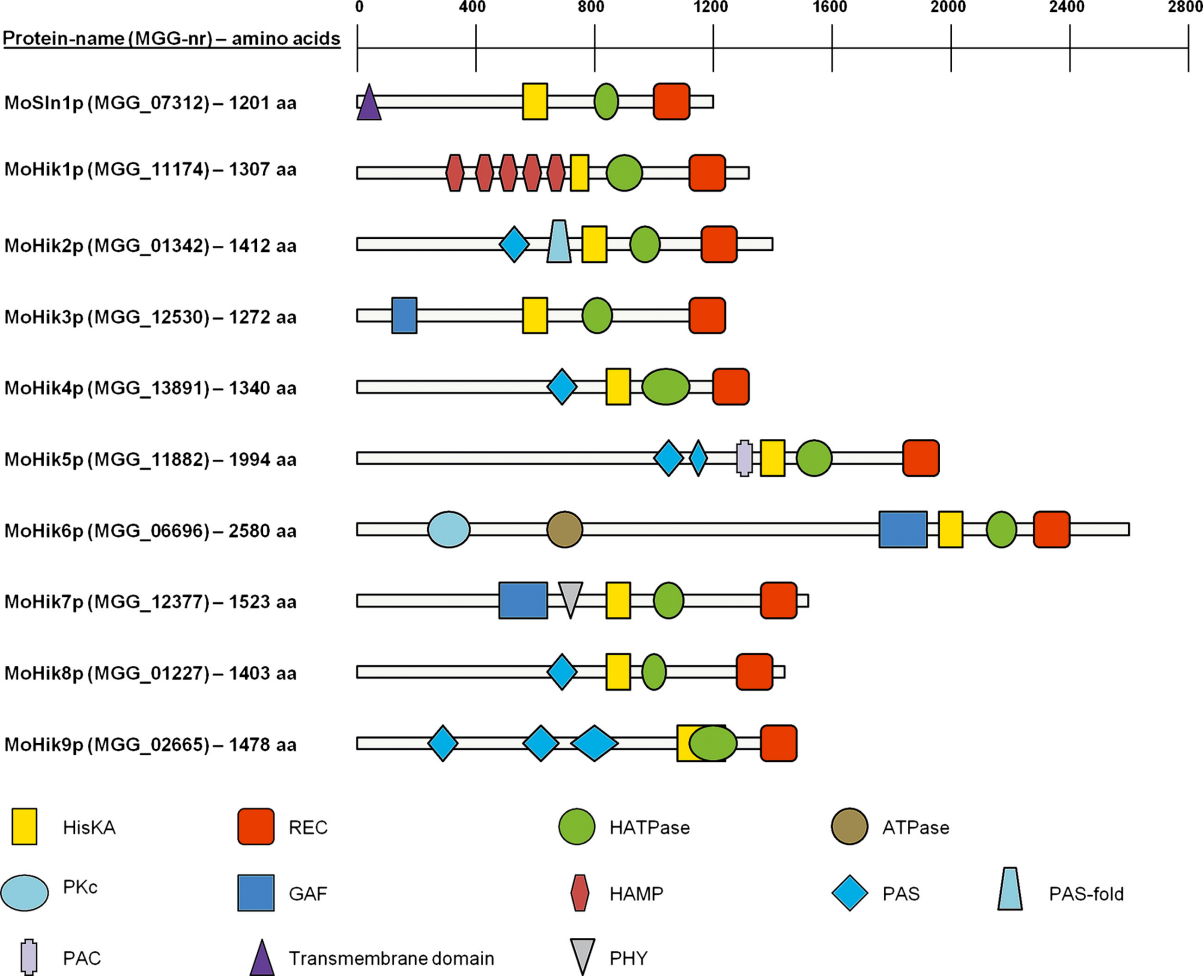
Schematic presentation of the protein domains of the 10 hybrid histidine kinases in *Magnaporthe oryzae*. For each protein, the correlating gene name from the *Magnaporthe* comparative database (*Magnaporthe* comparative Sequencing Project, Broad Institute of Harvard and MIT [http://www.broadinstitute.org/], annotation *M. oryzae* 70-15 [MG8]) is shown in brackets followed by the protein length (aa). Specific conserved protein domains which were predicted using the CDD database of NCBI (http://www.ncbi.nlm.nih.gov/Structure/cdd/wrpsb.cgiand). The prediction of transmembrane helices was performed using the TMHMM Server v. 2.0 (http://www.cbs.dtu.dk/services/TMHMM-2.0/). HisKA, histidine kinase domain; REC, regulatory domain; HATPase, histidine-ATPase domain; ATPase, ATPase domain; PKc, protein kinase domain; GAF, GAF domain; HAMP, HAMP domain; PAS, PAS domain; PASF, PASF domain; PAC, PAC domain; PHY, phytochrome domain; TM, transmembrane domain.

In order to obtain a comprehensive overview of HIK protein functions, all genes encoding sequences with a conserved HisKA (histidine kinase A, phosphoacceptor)-signaling domain (PF00512, http://www.ncbi.nlm.nih.gov/Structure/cdd/wrpsb.cgi; CDD database NCBI) within the *M. oryzae* genome were identified (Fig.2). In addition to the previously described *MoSLN1* (MGG_07312; Zhang et al. 2010) and *MoHIK1* (MGG_11174; Motoyama et al. 2005a), the newly found histidine kinase-encoding genes were named *MoHIK2* (MGG_01342), *MoHIK3* (MGG_12530), *MoHIK4* (MGG_13891), *MoHIK5* (MGG_11882), *MoHIK6* (MGG_06696), *MoHIK7* (MGG_12377), *MoHIK8* (MGG_01227), and *MoHIK9* (MGG_02665). The corresponding proteins to these genes contain in addition to the HisKA domain, a REC (signal receiver) domain (PF00072), a HATPase (histidine-like ATPase; PF02518) domain, and different signaling domains, for example, GAF- (cGMP-specific phosphodiesterases, adenylyl cyclases and FhlA [formate hydrogen lyase transcriptional activator in *E. coli*]; PF01590), PHY- (phytochrome region; PF01590), PKc- (protein kinases, catalytic domain; cd00180), HAMP- (histidine kinase, adenylyl cyclase, methyl-accepting protein, and phosphatase; PF00672), PAS- (period circadian protein, aryl hydrocarbon receptor nuclear translocator protein, single-minded protein; PF00989), PAS-fold- (PF08448), PAC- (motif C-terminal to PAS-motifs; smart00086), and ATPase domains (PF13191) (Fig.2). However, we were unable to identify transmembrane helices for MoHik1p–MoHik9p. Only one *N*-terminal transmembane helix (amino acids 9–31) was predicted in the sequence of MoSln1p. Analysis of transmembrane segments with the prediction tool “DAS” transmembrane prediction server suggests a second possible transmembrane domain at position 394–416 of the amino acid sequence (data not shown, also published by Zhang et al. 2010). Nevertheless, the score of this second domain implies that it is not suitable for a significant proposition. All other sensor HIK proteins appear to be located in the cytosolic space or at most associated to the membrane.

### Phylogenetic analysis of HIKs in fungi

**Figure 3:**
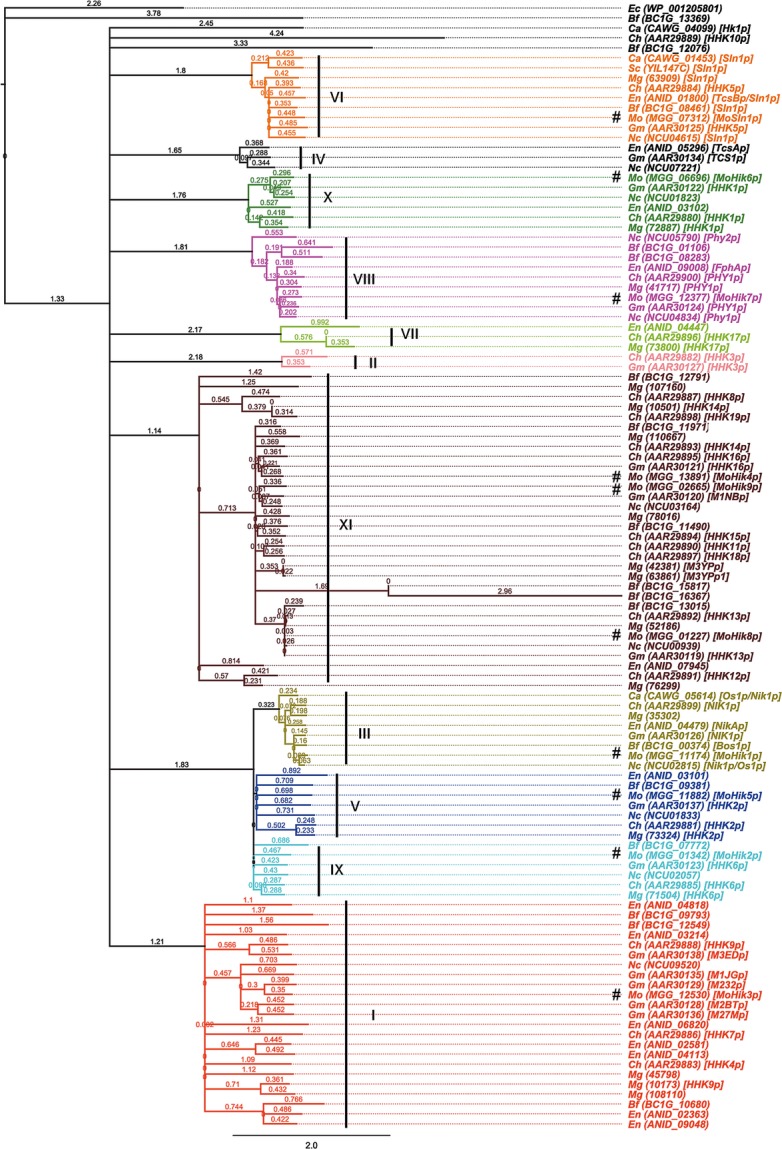
Phylogenetic analysis of amino acid sequences of HIKs from *Magnaporthe oryzae* and selected fungal species. The GeneBank accession numbers of the analyzed proteins or the gene name from the *Magnaporthe* comparative database are shown in curved brackets and trivial names were shown in square brackets. A preceding hash tags the protein sequences of *M. oryzae*. Related clades of the dendrogram have the same color and group number (roman numerals, according to Catlett et al. 2003). Bf (*Botryotinia fuckeliana*), Ca (*Candida albicans*), Ch (*Cochliobolus heterostrophus*), Ec (*Escherichia coli*), En (*Emericella nidulans*), Gm (*Gibberella moniliformis*), Mg (*Mycosphaerella graminicola*), Mo (*Magnaporthe oryzae*), Nc (*Neurospora crassa*), Sc (*Saccharomyces cerevisiae*). HIK, histidine kinases.

Two component sensor hybrid HIKs in fungi were categorized in 11 groups (Catlett et al. 2003). Amino acid sequences of the 10 sensor HIK proteins of *M. oryzae* and, additionally, 103 HIK-encoding sequences of selected fungi were analyzed resulting in a phylogenetic tree visualizing the different groups. We selected *Botryotinia fuckeliana* (anamorph: *Botrytis cinerea*, Leotiomycetes) (17 HIKs), *Candida albicans* (Saccharomycetes) (3 HIKs), *Cochliobolus heterostrophus* (*Bipolaris maydis*, Dothideomycetes) (21 HIKs), *Emericella nidulans* (*Aspergillus nidulans*, Plectomycetes) (15 HIKs), *Gibberella moniliformis* (*Fusarium verticillioides*, Sordariomycetes) (16 HIKs), *Mycosphaerella graminicola* (*Zymoseptoria tritici*, Dothideomycetes) (19 HIKs), *Neurospora crassa* (*Crysolinia crassa*, Sordariomycetes) (11 HIKs), and *Saccharomyces cerevisiae* (*Candida robusta*, Saccharomycetes) (1 HIK). The analysis revealed the 11 major groups and except for groups II, IV, and VII we identified at least one member of *M. oryzae* in every group (Fig.3). For a list of the accession numbers, names, schematic presentation of the protein domains, and the databases we used, see the supporting information Table S3 and Figure S2.

### MoHik5p and MoHik8p are required for conidial morphology and appressorium formation in *M. oryzae*

**Figure 4:**
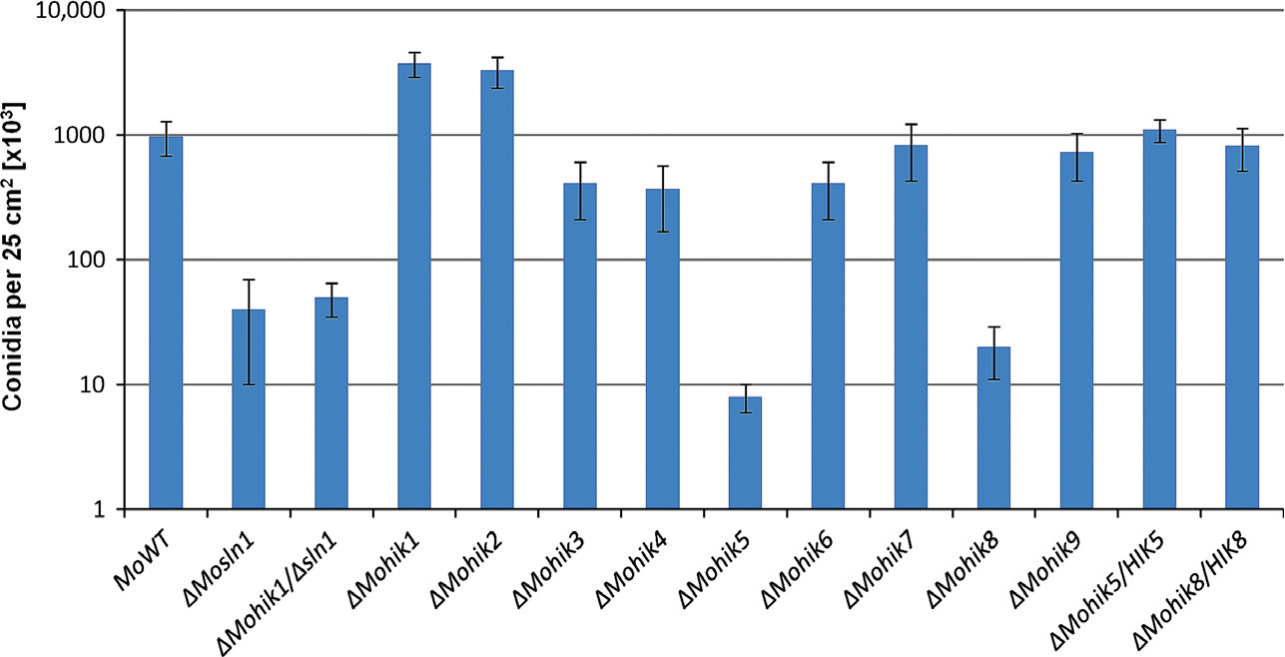
Conidia formation in *Magnaporthe oryzae* strain 70-15 and the HIK mutants. The number of conidia per 25 cm^2^ culture on solid medium is shown. The error bars represents the standard deviation of three experiments with five replicates each. HIK, histidine kinases.

The ability of the mutants to form conidia was assessed. A high variation in conidia production was observed within the mutant strains compared to the *M. oryzae* WT. *ΔMohik1* and *ΔMohik2* were found to produce slightly increased numbers of conidia, whereas the mutants *ΔMosln1*, *ΔMohik5*, *ΔMohik8* as well as the double mutant *ΔMohik1/Δsln1* are reduced in conidia production (Fig.4).

**Figure 5:**
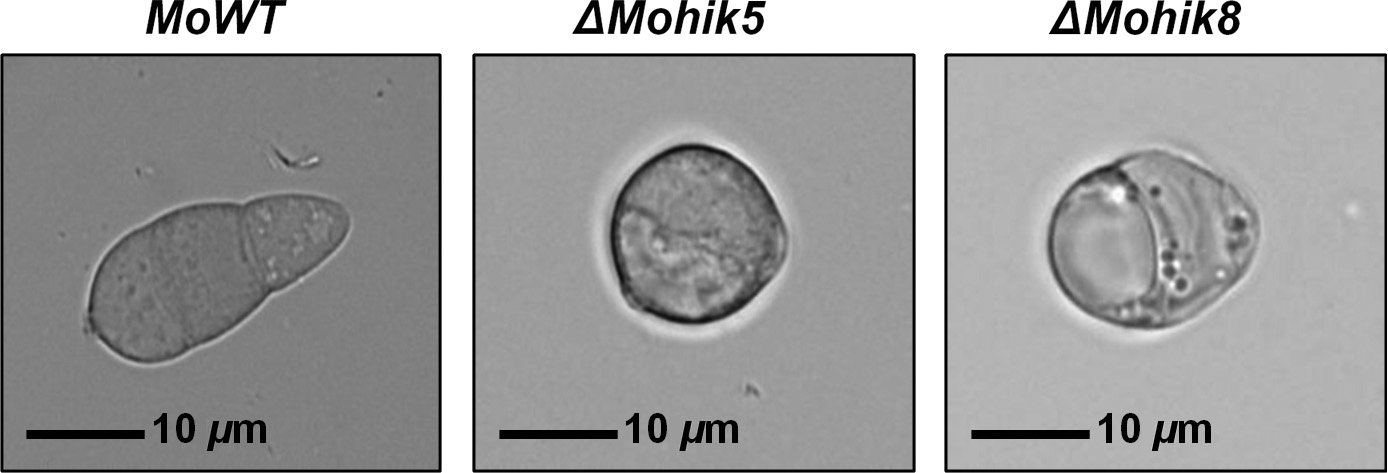
Conidia of the *Magnaporthe oryzae* strain 70-15 and the HIK mutants *ΔMohik5* and *ΔMohik8*. Conidia of *ΔMohik5* and *ΔMohik8* were 1- to 2 celled and almost round, whereas the wild-type conidia were typically three celled and elongated. The scale bar represents a length of 10 *μ*m. HIK, histidine kinases.

Microscopic analysis revealed abnormal conidial morphology in the mutant strains *ΔMohik5* and *ΔMohik8*. Conidia of the WT are typically three celled and ellipsoidal. The mutant strains *ΔMohik5* and *ΔMohik8* both produce 1–2 celled, round conidia (Fig.5). It was furthermore recorded that two germ tubes emerge on opposite ends of the conidia (Fig. 7). The complemented strains *ΔMohik5/HIK5* and *ΔMohik8/HIK8* were found to produce as many conidia as the WT and these were mostly three celled and ellipsoidal (data not shown).

**Figure 6:**
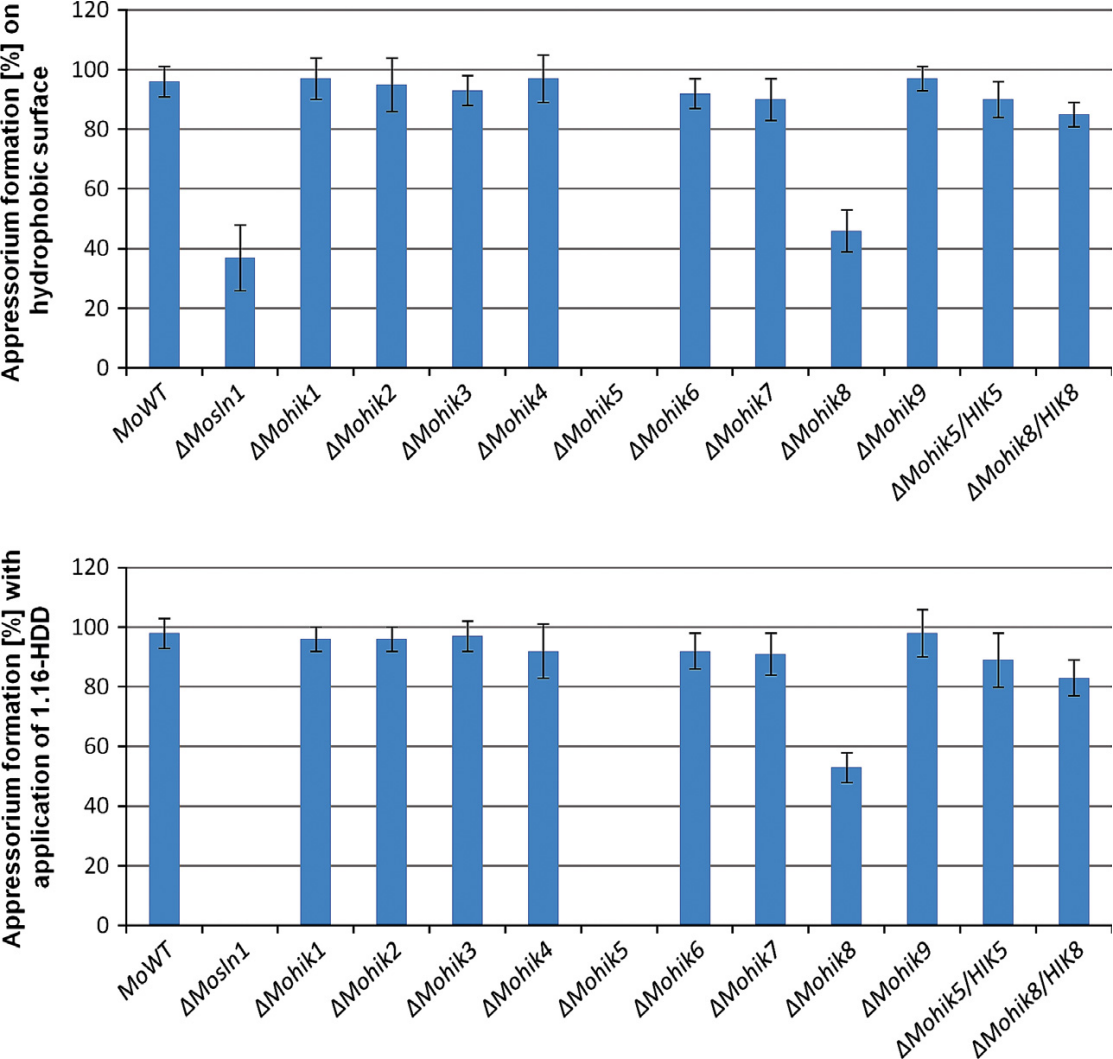
Appressorium formation of *Magnaporthe oryzae* strain 70-15 and the HIK mutants. The diagram shows the number of appressoria after an incubation period of 16 h at RT in H_2_O on a hydrophobic surface or induced by 1,16-HDD. The error bars represent the standard deviation of three experiments with five replicates each. HIK, histidine kinases.

Physical cues of an inductive surface are known to be required for appressorium formation in *M. oryzae* (Lee and Dean 1994). The ability of the mutants to form the infection structures was monitored on artificial hydrophobic surfaces (plastic slides) and on hydrophilic surfaces (glass cover slides) upon application of 1,16-HDD (Fig.6). The mutant strain *ΔMohik5* failed to develop the specialized infection structures in both assays. In case of the mutant strain *ΔMohik8*, a reduced number of deformed infection cells was recorded. The mutant strain *ΔMosln1* showed a reduced number of deformed infection structures on hydrophobic plastic slides. In contrast the strain failed to form infection structures after application of 1,16-HDD. The same phenomenon was observed in the *ΔMohik1/Δsln1* double mutant (data not shown).

**Figure 7:**
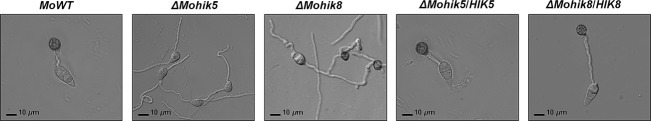
Appressoria of *Magnaporthe oryzae* strain 70-15, *ΔMohik5*, *ΔMohik8*, and the complemented strains *ΔMohik5/HIK5* and *ΔMohik8/HIK8* on hydrophobic plastic cover slips. The photographs were taken after an incubation period of 16 h at RT in H_2_O on a hydrophobic surface. HIK, histidine kinases.

The strains *ΔMohik5* and *ΔMohik8* were found to form longer germination hyphae as compared to the WT (Fig.7). Accordingly, functional MoHik5p and MoHik8p proteins are required for appropriate development of the infection structures and thus maybe necessary for successful penetration and colonization of the host plant. Reintegration of the genes *MoHIK5* and *MoHIK8* into the mutant strains *ΔMohik5* and *ΔMohik8*, respectively, complemented these effects (Fig.7).

### MoSln1p, MoHik5p, and MoHik9p are essential for cell wall stability

**Figure 8:**
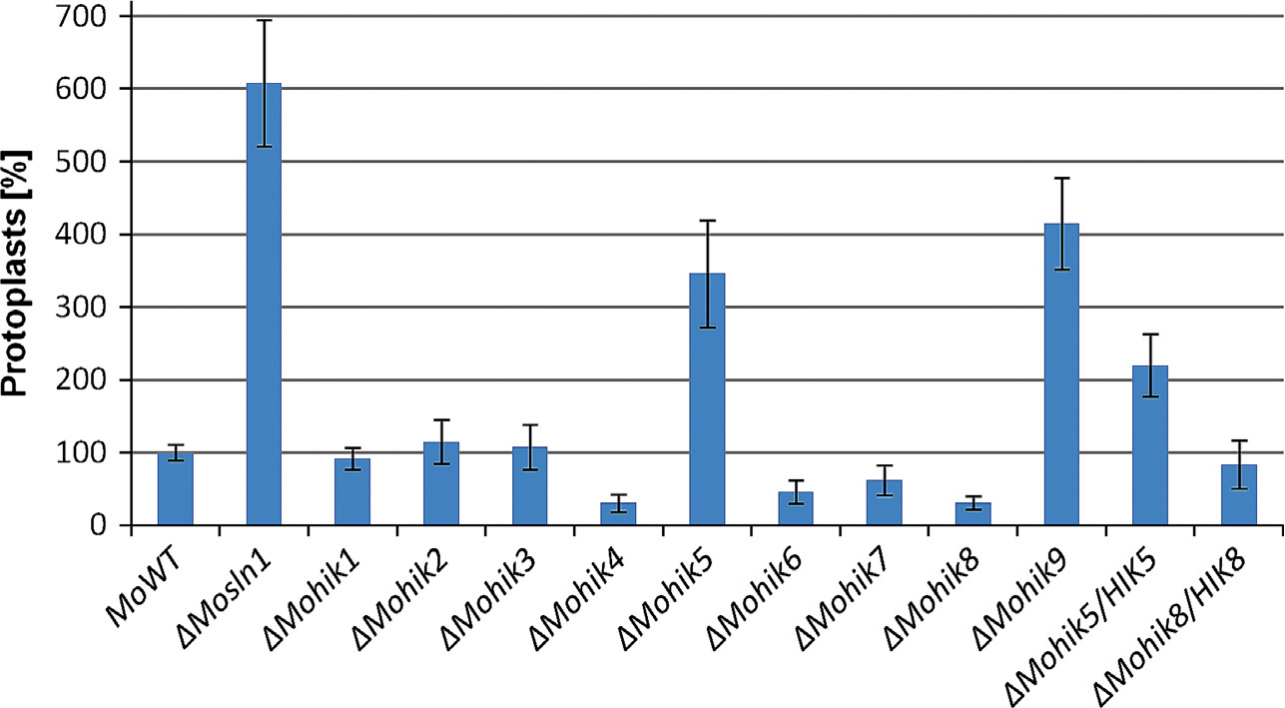
Cell wall stability of mycelia from the *Magnaporthe oryzae* wild-type strain 70-15 and the HIK mutants. The diagram shows the number of protoplasts after an incubation period of 1 h at RT in 3 mg/mL lysing enzymes from *Trichoderma harzianum* in 20% sucrose solution. The error bars represent the standard deviation of three experiments with five replicates each. HIK, histidine kinases.

Inactivation of *MoSLN1*, *MoHIK5*, and *MoHIK9* led to a defect in cell wall stability toward cell wall degrading lysing enzymes from *Trichoderma harzianum*. After 60 min incubation with lysing enzymes, the number of spheroplasts in the samples of the mutant strain *ΔMosln1*, *ΔMohik5*, and *ΔMohik9* was found to be three- to sixfold higher in contrast to the WT (Fig.8). The mutants show hypersensitivity to lysing enzymes. However, hyphae of the mutant strains *ΔMohik4*, *ΔMohik6*, and *ΔMohik8* were found to be more resistant toward lytic enzymes compared to the WT (Fig.8).

### HIKs are involved in stress signaling and in adaption to environmental changes

In order to investigate whether the inactivation of HIKs affect vegetative growth in *M. oryzae*, we monitored growth rates of WT and the mutant strains in different media. After 10 days incubation on media containing different stress inducing ingredients, such as NaCl (salt stress) and NaNO_2_ (hypoxia/nitrosative stress), sorbitol (osmotic stress), CoCl_2_ (hypoxia stress), CuSO_4_ (ion stress), and H_2_O_2_ (ROS, reactive oxygen species), growth rates were measured. A statistical quantification and analysis of the radial growth rate/diameter are given in Tables2 and S2. Compared to the *M. oryzae* WT the mutant strains *ΔMosln1*, *ΔMohik5*, and *ΔMohik8* growth rates were decreased on CM as well as on MM. It was found that vegetative growth of *ΔMohik5* was affected most significantly (see also Figs. S3 and S4).

**Table 2:** Vegetative growth of the *Magnaporthe oryzae* wild-type strain 70-15 and the HIK mutants (normalized results from Table S2).

	*MoWT*	*ΔMosln1*	*ΔMohik1*	*ΔMohik1/Δsln1*	*ΔMohik2*	*ΔMohik3*	*ΔMohik4*	*ΔMohik5*	*ΔMohik6*	*ΔMohik7*	*ΔMohik8*	*ΔMohik9*	*ΔMohik5/HIK5*	*ΔMohik8/HIK8*
Relative colony diameter (%)														
CM	100	100	100	100	100	100	100	100	100	100	100	100	100	100
CM + 0.8 mol/L NaCl	59	45	53	14	49	58	61	51	53	61	60	49	69	61
CM + 1 mol/L sorbitol	67	68	22	18	58	64	65	71	64	63	61	58	67	59
CM + 0.2 mol/L NaNO_2_	58	17	35	14	29	41	0	24	59	55	48	21	46	61
CM + 1 mmol/L CoCl_2_	57	65	38	63	0	34	35	20	53	43	16	23	46	30
CM + 4 mmol/L CuSO_4_	61	62	64	57	48	58	63	61	57	58	61	63	70	59
CM 32°C	82	69	71	72	93	93	76	90	95	89	92	82	96	80
MM	100	100	100	100	100	100	100	100	100	100	100	100	100	100
MM + 0.5 mol/L NaCl	43	0	43	0	53	42	38	53	37	42	35	43	39	42
MM + 0.8 mol/L sorbitol	78	80	36	27	77	75	65	78	79	75	71	76	57	82
MM + 0.1 mol/L NaNO_2_	63	0	50	0	73	51	47	40	21	61	63	0	67	63
MM + 1 mmol/L CoCl_2_	56	49	31	0	30	52	36	27	44	36	17	30	33	60
MM + 2 mmol/L CuSO_4_	93	75	84	101	85	86	68	62	77	82	81	63	66	90
MM 32°C	75	74	79	70	83	77	74	98	76	74	98	76	72	79
MM + 5 mmol/L H_2_O_2_	76	67	43	55	59	70	76	40	79	69	46	34	64	69

The vegetative growth assays were conducted on complete medium (CM) and on minimal medium (MM), and on CM and MM inclusive NaCl, sorbitol, NaNO_2_, CoCl_2_, CuSO_4_, H_2_O_2_ for 10 days at 26°C. In addition equivalent experiments were set up on CM and MM for 10 days at 32°C. The table shows the growth rates relative to 100% growth on CM or MM without stressing ingredients. The experiments were repeated three times with five replicates each.

**Figure 9:**
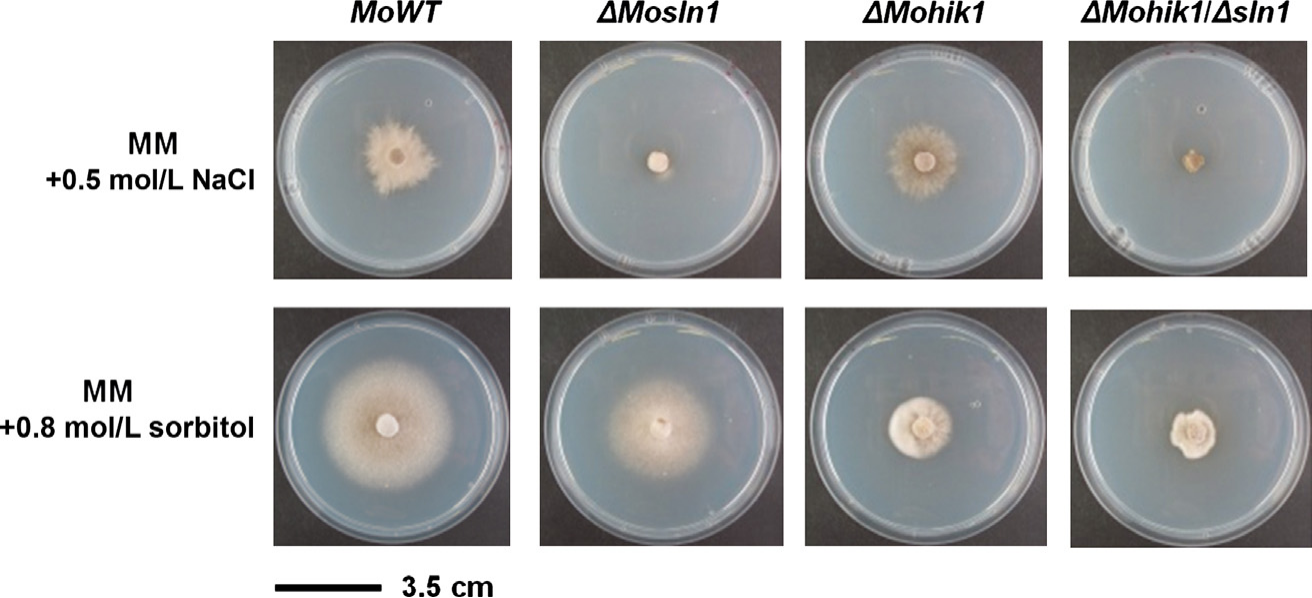
Vegetative growth of the *Magnaporthe oryzae* wild-type strain 70-15 and *ΔMosln1*, *ΔMohik1*, and *ΔMohik1/Δsln1*. The fungal colonies were grown on minimal medium (MM) with additional stress inducing agents NaCl or sorbitol for 10 days at 26°C.

Salt stress (NaCl) and osmotic stress (sorbitol) are influences to which the fungus is exposed during plant colonization. The degradation of plant tissue due to the colonization by the pathogen results in changes of the environmental conditions for the fungus. In order to investigate the role of the HIKs under such conditions WT and mutant strains were exposed to either salt stress (NaCl) or osmotic stress (sorbitol). In CM, the ingredients affect *ΔMosln1*, *ΔMohik1*, *ΔMohik2*, and *ΔMohik9* more significantly compared to the WT. In contrast, growth rates in MM were not as significantly altered as in CM (Table2; Figs. S3 and S4). We found that *ΔMosln1* is strongly influenced by salt stress compared to *ΔMohik1*, whereas *ΔMohik1* is more sensitive to osmotic or sugar stress (Fig.9). Interestingly the double mutant *ΔMohik1/Δsln1* failed to grow on both of the media either under salt or osmotic stress. That indicates the overlapping function of these two HIKs in reaction to salt or sugar stress via the HOG-signaling pathway. Additionally, the same phenomenon for the double mutant *ΔMohik1*/*Δsln1* was observed on media including NaNO_2_ and CoCl_2_ (Table2; Figs. S3 and S4) as well as on media including KCl, NaNO_3_, and (NH_4_)_2_SO_4_ (data not shown).

NaNO_3_, (NH_4_)_2_SO_4_ (data not shown), and NaNO_2_ were used to assess whether the HIKs have a function in nitrogen metabolism, detoxification, or uptake in *M. oryzae*. It was found that nitrate has no effect on viability of the mutant strains except for *ΔMohik1/Δsln1*. In contrast to the WT and the other mutants, the double mutant is highly sensitive to nitrate stress (data not shown). Nitrite has strong effects on the viability of the mutants. On CM, all mutants except for *ΔMohik3*, *ΔMohik6*, *ΔMohik7*, and *ΔMohik8* are strongly influenced concerning growth by NaNO_2_ (Table2; Fig. S3). However, in MM the results concerning the influence on vegetative growth differed significantly. Whether the mutants *ΔMohik2*, *ΔMohik7*, and *ΔMohik8* show a growth rate similar to the WT, the mutant strains *ΔMosln1*, *ΔMohik5*, and *ΔMohik9* were the most susceptible strains when exposed to nitrite stress (Table2; Fig. S4).

The hypoxia mimicking agent CoCl_2_ strongly influences mutants lacking signal HIKs. All mutants generated show reduced growth ability complemented by CoCl_2_. Especially the mutants lacking HIKs which contain oxygen sensing PAS domains (*ΔMohik2*, *ΔMohik4*, *ΔMohik5*, *ΔMohik8*, and *ΔMohik9*) were affected in vegetative growth in a significant manner. Concerning vegetative growth, the mutants *ΔMosln1*, *ΔMohik5*, and *ΔMohik9* were the most susceptible strains under hypoxia conditions (Table2; Figs. S3 and S4).

Ion stress induced by CuSO_4_ resulted in dark mycelial pigmentation in the mutants *ΔMosln1*, *ΔMohik1/Δsln1*, *ΔMohik2*, and *ΔMohik5* on CM. MM including CuSO_4_ impaired the growth rate of *ΔMosln1*, *ΔMohik4*, *ΔMohik5*, *ΔMohik8*, and *ΔMohik9*. Significant temperature sensitivity was found for *ΔMosln1*, *ΔMohik1*, and *ΔMohik1/Δsln1* on CM (Table2; Figs. S3 and S4).

In order to test whether the generated mutant strains are susceptible to the first plant defense response, we investigated their ability to adapt or react upon exposure to ROS in culture media. We observed that growth is significantly reduced in all the mutant strains except for *ΔMohik4*, *ΔMohik6*, and *ΔMohik7* (Table2; Figs. S3 and S4).

### Additional signaling proteins trigger the HOG pathway by MoHog1p phosphorylation

In order to determine whether MoHik1p, MoSln1p, or both are responsible for signal perception, western blot analysis of phospho-MoHog1p was used to detect the activation of the HOG-signaling pathway by salt stress (NaCl) and high osmolarity (sorbitol). The phosphorylation signal of MoHog1p MAPK was investigated using a western blot analysis with a phospho-p38 MAPK (Thr180/Tyr182) (D3F9) XP™ rabbit monoclonal antibody (Cell Signaling Technology). MoHog1p phosphorylation was detected in the WT treated with 0.5 mol/L/1.0 mol/L NaCl and 0.5 mol/L/1.0 mol/L sorbitol. In contrast, no detectible phosphorylation of MoHog1p was observed under identical conditions in the *ΔMohog1* mutant, thereby indicating that MoHog1p is activated under salt stress and high osmolarity. Surprisingly, we detected phosphorylation signals of MoHog1p in the mutant strains *ΔMohik1*, *ΔMosln1*, and *ΔMohik1/Δsln1* (Fig.10).

**Figure:**
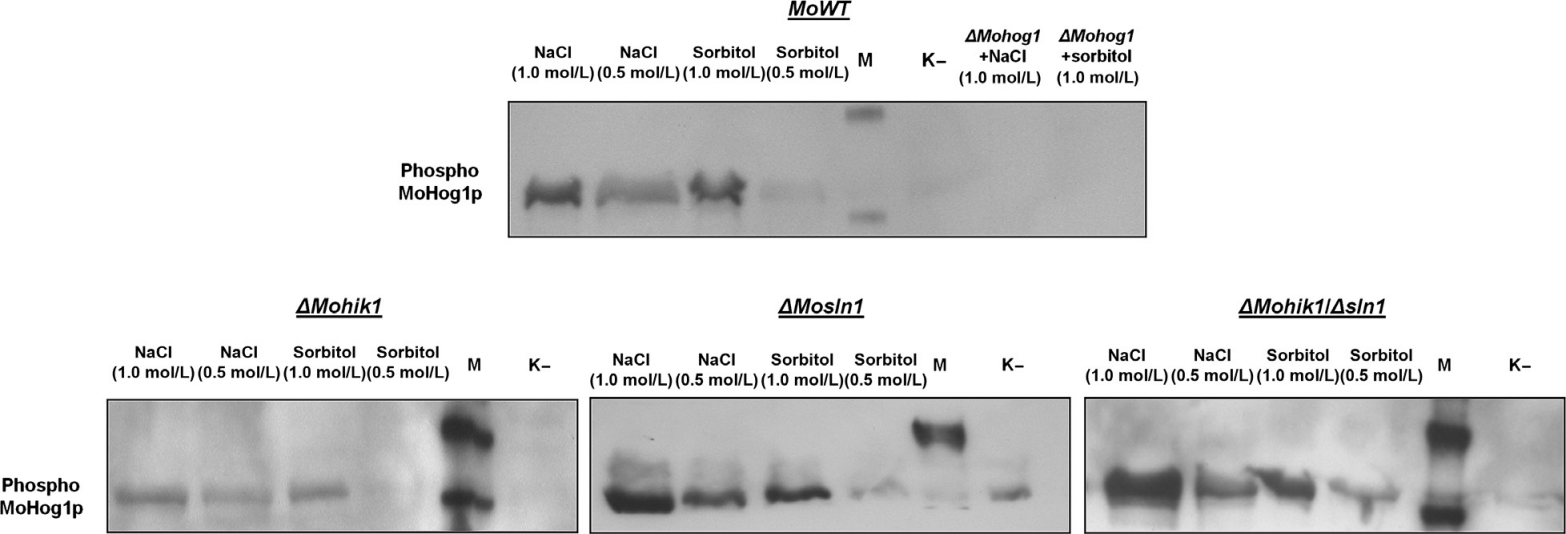
Phosphorylation of MoHog1p MAPK in the *Magnaporthe oryzae* wild-type strain 70-15 and in the mutant strains *ΔMohog1*, *ΔMohik1*, *ΔMosln1*, and *ΔMohik1/Δsln1*. Mycelia of the strains *M. oryzae* 70-15 and the gene inactivation mutants were used for western blot analysis as described in Experimental Procedures. Incubation in CM was used as negative control (K−). The WT and the gene inactivation mutants were incubated in CM with 0.5 mol/L/1.0 mol/L NaCl or 0.5 mol/L/1.0 mol/L sorbitol. M is the biotinylated protein ladder (Cell Signaling Technology, Beverly, MA).

Probably there are more sensor elements than MoHik1p and MoSln1p upstream MoSsk1p and the MAPK cascade MoSsk2p-MoPbs2p-MoHog1p in the signaling pathway responsible for activation of MoHog1p. If only MoHik1p or MoSln1p are responsible for detection of salt and osmotic stress, there should be no signal of phospho-MoHog1p in the mutant strains after application of NaCl or sorbitol. If MoHik1p and MoSln1p act together, there should be also no signal in the double mutant strain. However, our experiments show distinct phosphorylation signals in the WT and in all the mutant strains, including *ΔMohik1/Δsln1* (Fig.10).

### MoSln1p, MoHik5p, and MoHik9p in a model for sensing oxygen within the HOG pathway

**Figure 11:**
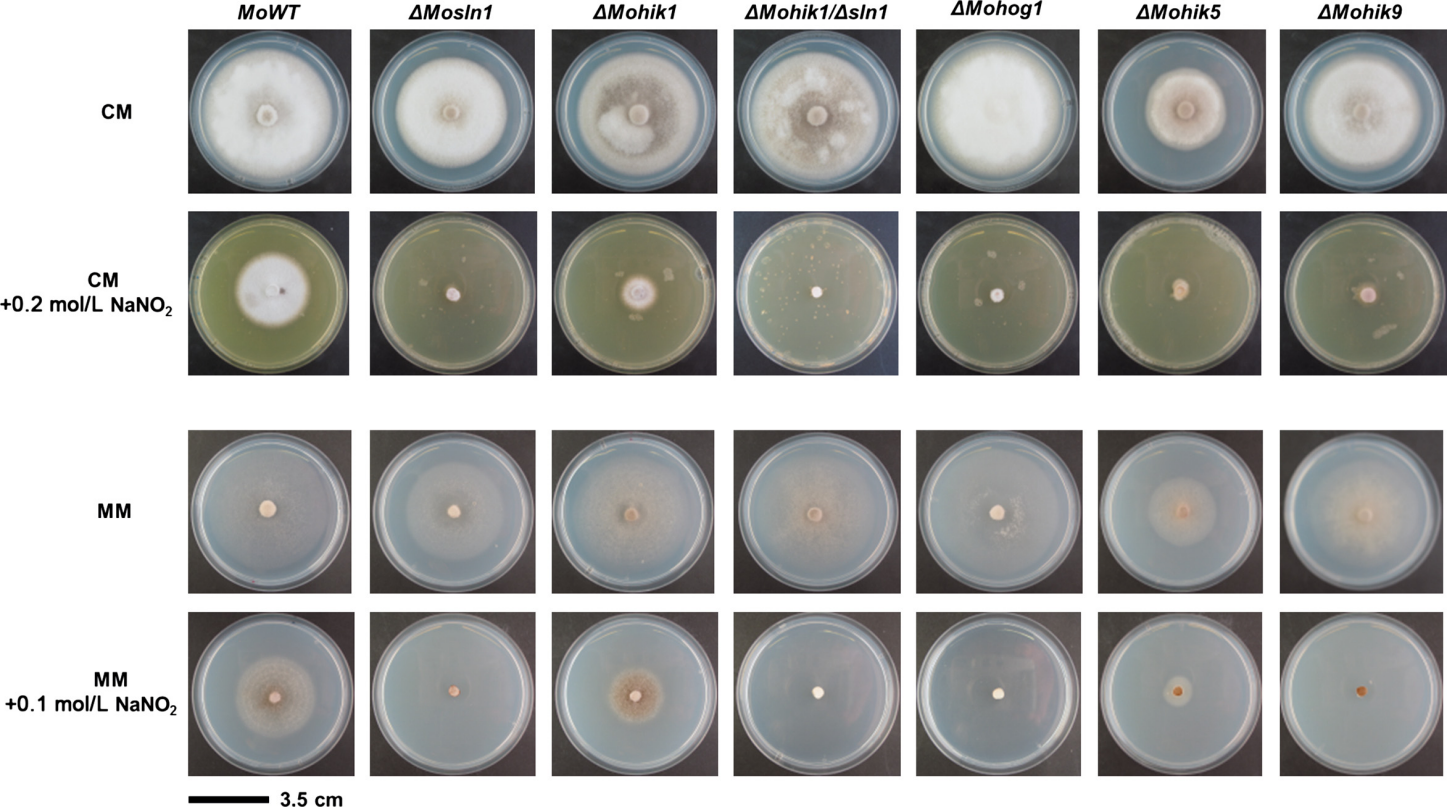
Vegetative growth of the *Magnaporthe oryzae* wild-type strain 70-15 and the HIK mutants under nitrite stress. The fungal colonies were grown on CM with additional stress inducing agent NaNO_2_ for 10 days at 26°C. HIK, histidine kinases.

The vegetative growth assays with NaNO_2_ indicated that the mutant strains *ΔMosln1*, *ΔMohik5*, and *ΔMohik9* were the most affected (Table2; Figs. S3 and S4). This significant susceptibility may be down to the fact that MoSln1p, MoHik5p, and MoHik9p act together within the HOG-signaling cascade. We therefore additionally monitored the sensitivity of the *ΔMohog1* mutant toward NaNO_2_ (Fig.11).

**Figure 12:**
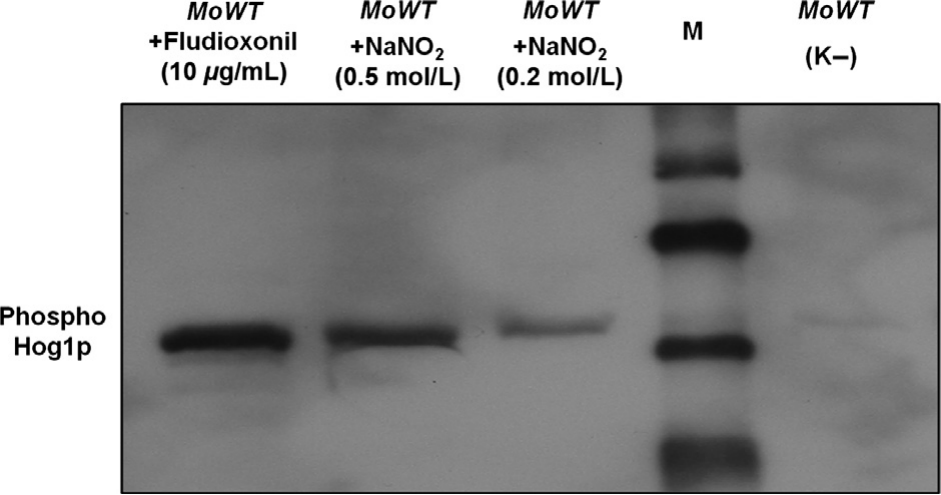
NaNO_2_-induced phosphorylation of MoHog1p MAPK in the *Magnaporthe oryzae* wild-type strain 70-15. Mycelium of the *M. oryzae* 70-15 strain was used for western blot analysis as described in Experimental Procedures. Incubation in CM was used as negative control (K−). The WT was incubated in CM with 0.2 mol/L/0.5 mol/L NaNO_2_ for 10 min at room temperature. M is the biotinylated protein ladder (Cell Signaling Technology, Beverly, MA).

The activation of the HOG-signaling pathway through NaNO_2_ was confirmed by western analysis (Fig.12). Distinct signals of phosphorylated MoHog1p were observed after exposure of the strains to hypoxia mimicking nitrite stress (0.2 mol/L or 0.5 mol/L NaNO_2_). These results suggest that parts of the HOG pathway have function in oxygen sensing in *M. oryzae*.

### HIKs are required for full virulence, MoHik5p and MoHik8p are pathogenicity factors

**Figure 13:**
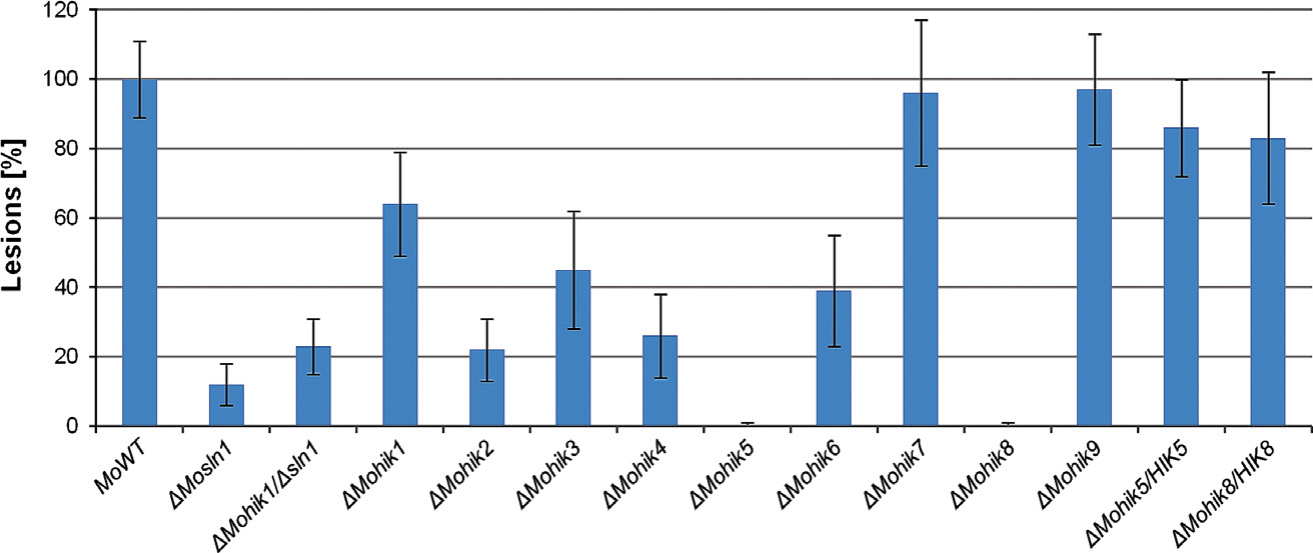
Pathogenicity-assay of the *Magnaporthe oryzae* wild-type strain 70-15 and the HIK mutants on rice plants. The plant infection assays were carried out as described in Experimental Procedures. The error bars represent the standard deviation of three experiments with five replicates each. HIK, histidine kinases.

To determine the role of HIKs in pathogenesis of *M. oryzae*, plant infection assays were carried out to investigate the ability of the mutants to infect rice plants. We previously identified abnormal conidial morphology in the mutant strains *ΔMohik5* and *ΔMohik8* (Fig.5) as well as abnormal, absent appressorium formation in *ΔMosln1*, *ΔMohik5*, and *ΔMohik8* (Fig.7). The virulence to susceptible rice cultivar CO-39 was significantly reduced in all the HIK-mutant strains except for *ΔMohik7* and *ΔMohik9* (Fig.13).

**Figure 14:**
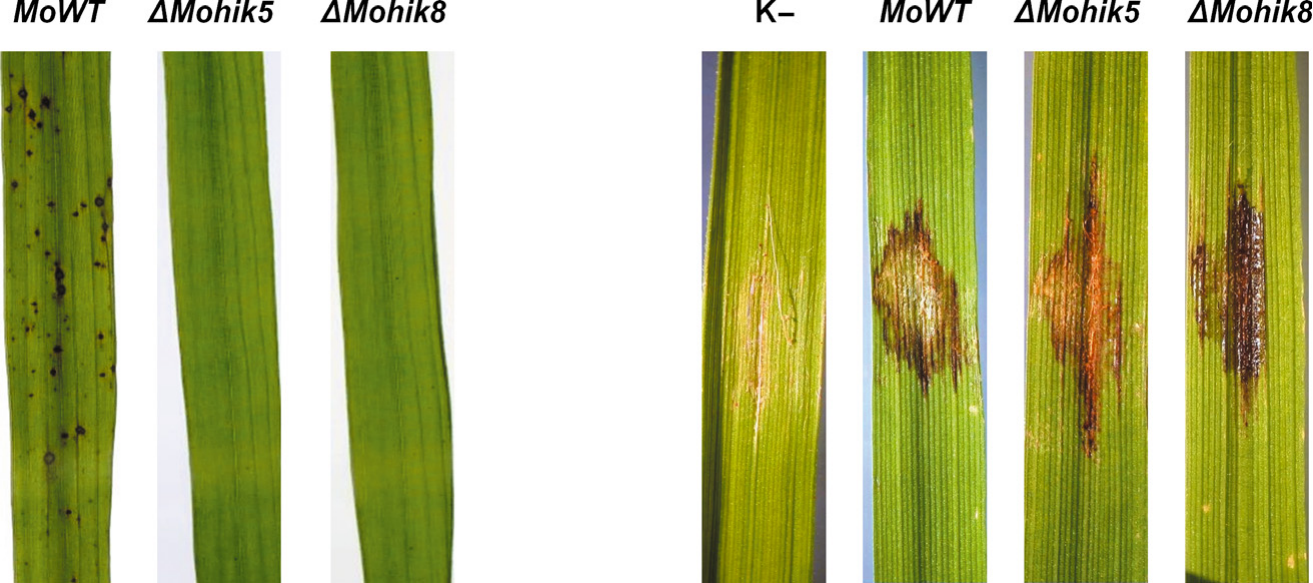
Rice leaves of cultivar CO-39 infected with the *Magnaporthe oryzae* wild-type strain 70-15 and the HIK mutants *ΔMohik5* and *ΔMohik8*. The plant infection assays (left) and studies of in planta growth (right) were carried out as described in Experimental Procedures. Negative controls were set up with H_2_O containing 0.2% gelatin. HIK, histidine kinases.

The number of lesions caused by the gene inactivation mutants were distinctly reduced as compared to the WT. *ΔMohik5* and *ΔMohik8* were found to be nonpathogenic, whereas the mutant strain *ΔMosln1* caused very occasionally lesions. The complemented strains *ΔMohik5/HIK5* and *ΔMohik8/HIK8* were found to be as pathogenic as the WT strain (Fig.3). Our results concerning in planta growth of *ΔMohik5* and *ΔMohik8* demonstrated that the colonization of plant tissue was not different in the mutants compared to the WT (Fig.14).

## Discussion

Histidine kinases are involved in environmental stress responses, pathogenicity, hyphal development, and sporulation (Li et al. 1998; Hohmann 2002; Nemecek et al. 2006; Viaud et al. 2006) as well as in differentiation processes, chemotaxis, secondary metabolite production and virulence-associated processes in plant and animal pathogens (Grebe and Stock 1999; Wolanin et al. 2002). HIKs of fungi are composed of a high functional diversity due to their complexity of the structure of different signaling domains combined with the regulation capacity of several regulatory domains (Bahn 2008).

Phylogenetic amino acid sequence analysis of HIKs of selected fungal species confirmed the classification of these kinases into 11 groups. For this purpose, we analyzed amino acid sequences of all HIK-encoding genes within the genomes of the plant pathogenic fungi *B. fuckeliana*, *C. heterostrophus*, *G. moniliformis*, *M. graminicola*, and *M. oryzae*. Additional the HIK-encoding sequences of the saprophytes *N. crassa* and *E. nidulans* as well as sequences of the saccharomycetes *C. albicans* and *S. cerevisiae* were included. The resulting phylogenetic tree showed high diversity between different groups (Fig.3). Members of the groups III, IV, and X were found to be highly conserved and these members may represent orthologue proteins evolved with similar functions. HIKs belonging to group I or XI were found to be highly diverse possibly due to the evolution of single gene families within these groups. The function of these highly diverse proteins is apparently not important for basic functions as with members of the groups III and VI, in which some of the proteins are indispensable for viability (Yoshimi et al. 2005). As previously reported (Vetcher et al. 2007), the sequence of MoHik1p was assigned to the group III and the sequence of MoSln1p to group VI (Zhang et al. 2010). The remaining HIKs of *M. oryzae* were classified into the different groups (Fig.3). The sequence of MoHik8p, a protein found to be essential for conidial development and thus for pathogenicity-relating morphogenesis, was in the group XI. Group XI comprises various proteins from phytopathogenic fungi, suggesting that group XI-HIKs may be associated with adaptability to different ecological niches (i.e., the change from ex planta to in planta growth). The pathogenicity factor MoHik5p was found to be a member of the group V, in which only one representative of each species was listed. That indicates an important role of members of group V for physiological processes, for example, during vegetative growth or pathogenicity.

Prokaryotic HIKs are typically transmembrane proteins with an extracellular sensing domain, whereas many eukaryotic HIKs are found in the cytosolic space (Catlett et al. 2003). Only for the protein MoSln1p one transmembrane domain was identified within the present study. All further HIKs identified in *M. oryzae* appear to be cytosolic or at most associated to the membrane. In most eukaryotes environmental signals are detected directly or indirectly by the external *N*-terminal domain of sensor kinases. The additional regulatory C-terminal REC domain of the HIKs as well as the output domains of the response regulator receiver proteins mediate diversity to phosphorelay systems and are distinct from other protein families which implements specific signal transduction pathways (Loomis et al. 1997). The extracellular localization of external *N*-terminal domains does not inevitably indicate that these domains are required for sensing. HIKs do not necessarily possess soluble extracellular ligands for an appropriate detection of environmental or physiological situations. An example is the bacterial osmosensing histidine kinase EnvZ of *E. coli*. EnvZ is an integral membrane protein with its putative sensing domain in the periplasmic space. Interestingly, removal of this domain does not disrupt osmosensing. That implies an osmosensing mechanism for EnvZ other than ligand binding (Leonardo and Forst 1996). Changing osmolarity affects membrane integrity and EnvZ is believed to interact subsequently with other membrane associated proteins. A related mechanism regulates osmosensing in *S. cerevisiae*. Within the yeast HIK Sln1p its integral membrane domain is an essential prerequisite for accurate signal transduction (Ostrander and Gorman 1999). However, it is still not completely understood how soluble HIKs implement their functions. They may couple to transmembrane receptors, separate binding proteins or small ligands such as amino acids, sugars, or other chemoattractants (Loomis et al. 1997). The absence of transmembrane domains in all HIKs except MoSln1p suggests that they transduce internal signals, for example, redox potentials or small ligand binding (i.e., with a PAS domain, Taylor and Zhulin 1999), or additional receptor proteins may receive extracellular signals and relay these signals to the intracellular HIKs (Catlett et al. 2003).

Initial results of the phenotypical analysis of the mutant strains revealed that *ΔMohik5* and *ΔMohik8* produce one- or two-celled spherical conidia compared to typically three-celled ellipsoidal WT conidia (Fig.5). Mutants with two-celled conidia have already been generated by REMI insertional mutagenesis, but with no effects on conidial viability (Balhadère et al. 1999). Further mutations affecting conidiation were identified by random chemical or insertional mutagenesis (Shi and Leung 1995; Shi et al. 1998). The *ΔMocon5* and *ΔMocon6* mutants are aconidial, the *ΔMocon1* and *ΔMocon2* strains are strongly reduced in the conidiation and produce misshapen conidia, whereas the *ΔMocon4* and *ΔMocon7* mutants produce abnormal conidia. In most cases, the affected *con-*gene has not yet been isolated or characterized. Exclusively *MoCON7* was found to encode a transcription factor which regulates the transcription of genes encoding products which influence the biogenesis of the *M. oryzae* cell wall (Odenbach et al. 2007).

In our study, the mutant strain *ΔMohik5* failed to form appressoria on artificial surface, even upon stimulation with 1,16-HDD. In case of the mutant strain *ΔMohik8* production of nonfunctional appressoria was observed. The mutant strain *ΔMosln1* showed a decreased number of deformed infection structures on hydrophobic surface and interestingly, after application of 1,16-HDD no infection cells could be observed (Fig.6). Mutants with absent appressoria where previously reported for *MoPMK1* (Xu and Hamer 1996) and thus interplay between the HIKs, the HOG pathway and the *PMK1* pathway may be conceivable in *M. oryzae*.

The application of lytic enzymes revealed that cell wall stability was significantly decreased in the mutants *ΔMosln1*, *ΔMohik5*, and *ΔMohik9* (Fig.8). This phenomenon has previously been reported for the inactivation of *MoMPS1* (Xu et al. 1998) and *MoMCK1* (Jeon et al. 2008), supporting involvement of proteins contributing to cell wall stability. MoMps1p and MoMck1p are homologs of *S. cerevisiae* Slt2p and Bck1p proteins required for cell wall integrity. Maintaining cell wall integrity is a basic mechanism for pathogens to protect themselves against host defense.

**Figure 15:**
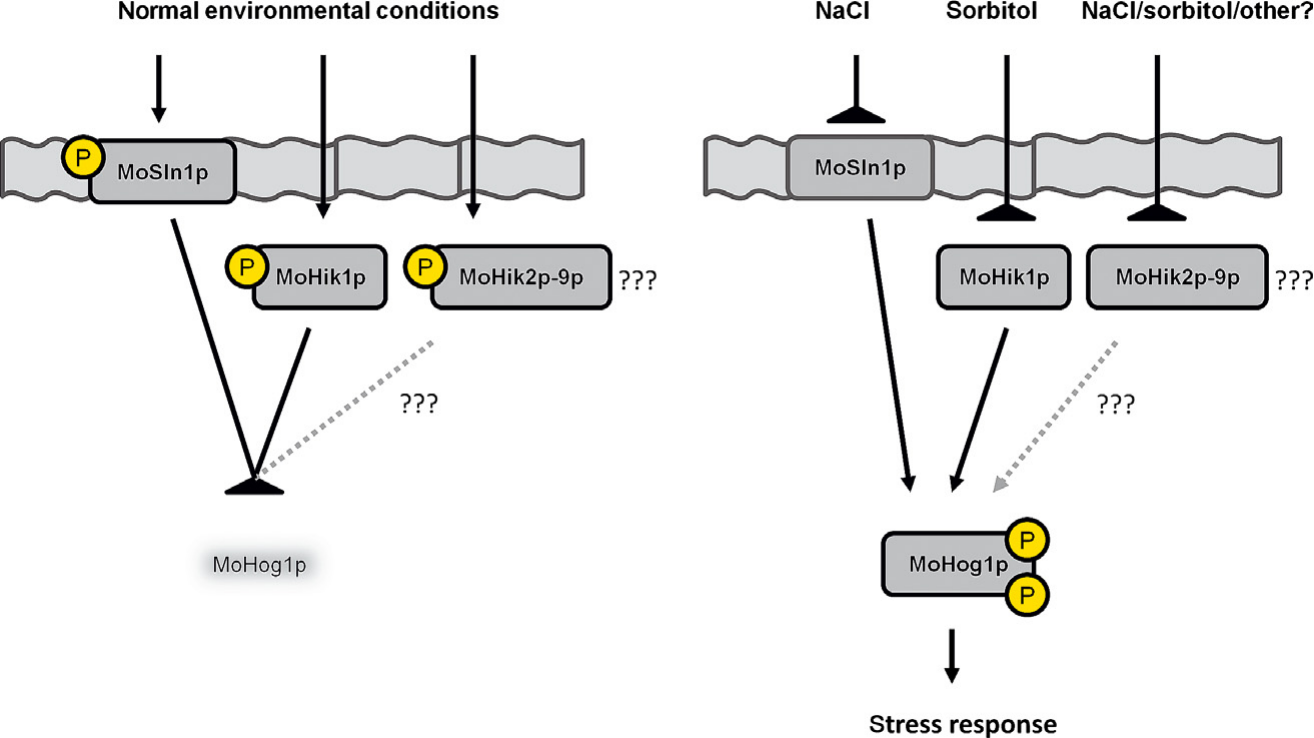
Simplified signal transduction scheme of salt and osmotic stress in *Magnaporthe oryzae* via the HOG pathway. Two hybrid histidine kinases (MoSln1p and MoHik1p) function as signal sensors for salt and high osmolarity. High osmolarity and salt stress result in inhibition of the sensor kinases and subsequently phosphorylation and activation of MoHog1p, which thus initiates the stress response.

MoSln1p and MoHik1p are both required for an appropriate cellular response to osmotic and salt stress in *M. oryzae* (Motoyama et al. 2008; Zhang et al. 2010). Our growth experiments show that the mutant *ΔMosln1* is more susceptible to salt stress (NaCl) compared to *ΔMohik1*, whereas *ΔMohik1* is more sensitive osmotic or sugar stress (sorbitol) in contrast to *ΔMosln1*. The double mutant *ΔMohik1/Δsln1* failed to grow under both conditions (Fig.1), indicating the overlapping function of these two HIKs in reaction to salt or sugar stress via the HOG pathway. MoSln1p appears to assume the physiological function of MoHik1p in the mutant *ΔMohik1* under salt or sugar stress and MoHik1p compensated MoSln1p in the mutant *ΔMosln1*. After inactivation of both HIK-encoding genes (*MoHIK1* and *MoSLN1*), we found that the HOG-signaling cascade is not completely inactivated. These findings are supported by western blot analysis of phosphorylated MoHog1p under salt and sugar stress. We observed that NaCl as well as sorbitol activated the HOG pathway due to phosphorylation of MoHog1p either in *ΔMosln1*, *ΔMohik1*, or in the *ΔMohik1/Δsln1* mutant (Fig.10). High osmolarity has to be mediated via additional sensor proteins. Maybe one or several of the remaining HIKs play additional roles in stress signaling in *M. oryzae* (Fig.15).

This hypothesis is supported by the finding that inactivation of *Mosln1*, *Mohik1* as well as double inactivation of both HIK-encoding genes is not lethal in *M. oryzae* in contrast to *S. cerevisiae*. Inactivation of the gene *SLN1* in *S. cerevisiae* constitutively activates the MAPK Hog1p, which is detrimental to the fungus (Maeda et al. 1994). Reports concerning the filamentous fungus *Cochliobolus heterostrophus* in which notably the Hog1-type MAPK is activated at high concentrations of osmolytes even in HIK deletion mutants furthermore support these findings (Yoshimi et al. 2005). Further components have to be identified and their function in this signaling cascade has to be studied. It is possible that various proteins or signaling pathways network together in sensing environmental stimuli. For example, in the basidiomycetous fungal pathogen *Cryptococcus neoformans*, cell toxicity via the HOG pathway caused by fludioxonil is enhanced by simultaneous inhibition of the cell integrity pathway (Kojima et al. 2006). It appears that an intensive crosstalk of different signaling pathways interact in pathogenic fungi.

Necrotrophic pathogens killing host tissues are expected to use a broader spectrum of nitrogen sources in contrast to a biotrophic pathogen which nourishes on living plant tissue with access to nitrogen sources exclusively available in the apoplast or the haustorial matrix (Snoeijers et al. 2000). Nitrite is a toxic intermediate of the reducing utilization of environmental nitrate to ammonia and has to be processed quickly. This extends to ammonia, which is directly used in physiological processes, for example, in the biosynthesis of glutamine (Marzluf 1997). The amide of glutamine is the nitrogen source for the synthesis of amino acids, purine and pyrimidine nucleotides, amino sugars, and coenzymes (Meister 1980). Nitrate has no effect on viability of the mutant strains except for *ΔMohik1/Δsln1*, which is highly sensitive to nitrate stress (data not shown).

**Figure16:**
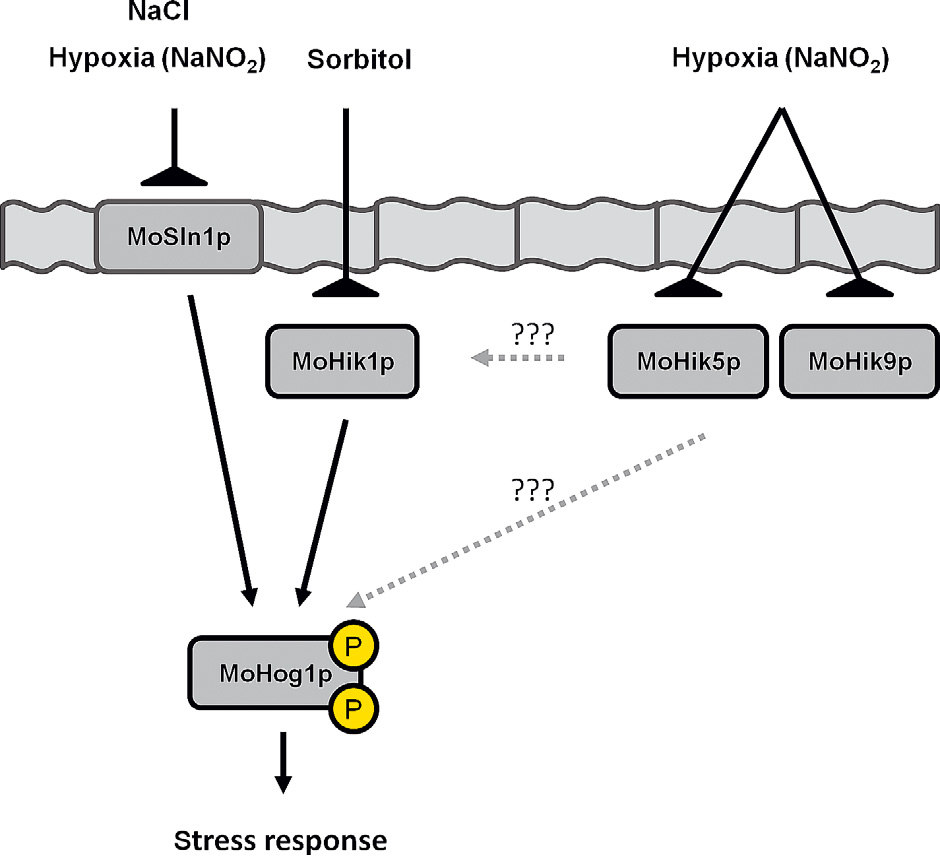
Simplified signal transduction scheme of NaNO_2_ in *Magnaporthe oryzae* via the HOG pathway. The hybrid histidine kinases MoSln1p, MoHik1p, MoHik5p, and MoHik9p function as signal sensors for NaNO_2_. Nitrite stress (low oxygen levels) causes inhibition of the sensor kinases resulting in phosphorylation and activation of MoHog1p.

MoSln1p, MoHik5p, and MoHik9p are involved in regulation of oxygen signaling, nitrite metabolism, or nitrite detoxification. The mutant strains *ΔMosln1*, *ΔMohik5*, *ΔMohik9* were the most susceptible strains to CoCl_2_ and NaNO_2_ in the growth assays (Table2; Figs. S3–S4). The results of the vegetative growth assays of the mutant strains *ΔMosln1*, *ΔMohik5*, *ΔMohik9*, and *ΔMohog1* under hypoxia-inducing CoCl_2_ or NaNO_2_ (Fig.1) and the experiments concerning cell wall stability of the same mutants (Fig.8) resulted in a proposed link between HIKs, HOG signaling, and oxygen sensing in *M. oryzae*. There were two to three PAS domains in the amino acid sequences of MoHik5p and MoHik9p, respectively, and PAS domains are known to mediate oxygen sensing in bacteria and fungi (Pellequer et al. 1999; Taylor and Zhulin 1999). Western analysis of phosphorylated MoHog1p confirmed MoHog1p-activation due to low oxygen levels induced by NaNO_2_ (Fig.2). NaNO_2_ is reduced within the cell to NO and reactive nitrogen species (RNS) have several targets within living cells which result in physiological reactions similar to hypoxia. RNS can affect iron- and sulfur centers of oxygen-dependent enzymes as well as DNA, sulfhydryl groups or lipids (De Groote and Fang 1995). Furthermore, RNS were found to inhibit enzymes of the respiratory chain (Joseph-Horne et al. 2001). MoSln1p, MoHik5p, and MoHik9p may be involved in sensing hypoxia, or transduce signals or processes which were triggered through hypoxia, at least, partially via the HOG pathway. With these results we were able to reveal a first simplified model for sensing low oxygen levels in *M. oryzae* (Fig.16).

Changing oxygen levels inside the plant tissue may influence the vitality of the fungus during the in planta growth phase. We propose that the PAS-domain containing proteins MoHik2p, MoHik4p, MoHik5p, and MoHik8p are essential for full virulence. An adequate reaction in the presence of ROS as the first plant defense reaction (Wojtaszek 1997) is necessary for full virulence of the fungal plant pathogen. All mutant strains reduced in virulence (except *ΔMohik4* and *ΔMohik6*) show also increased sensitivity to H_2_O_2_ in the growth assays (Table2; Fig. S4).

MoHik5p and MoHik8p appear to act as pathogenicity factors. The mutant strains *ΔMohik5* and *ΔMohik8* are completely nonpathogenic, whereas all other HIK-gene inactivation mutants except for *ΔMohik1*, *ΔMohik7*, and *ΔMohik9* are strong reduced in virulence (Fig.3). In planta growth of the mutants *ΔMohik5* and *ΔMohik8* was not affected (Fig.4). We assume the loss of pathogenicity of *ΔMohik5* and *ΔMohik8* is based on nonfunctional, absent appressoria and consequently the incapability to penetrate the plant cuticle, respectively.

Magnaporthe *oryzae* evolved conserved elements as well as unique signaling mechanisms for stress responses, virulence regulation, and morphological differentiation processes (Xu and Hamer 1996; Dixon et al. 1999). Therefore, this study provides insights into functions of virulence-associated HIKs within the rice blast fungus and furthermore contributes to the general understanding of important signaling pathways, that is, the HOG-signaling cascade. Due to their function within the same pathway, the proteins MoSln1p, MoHik5p, and MoHik8p are suggested to be good candidates for new fungicide targets. In case the pathogenicity factors are druggable, they may prove to be targets for protective plant protection as attractive alternatives for modern plant protection.

## Supporting Information

**Figure S1.** Southern blot analysis of gene inactivation mutants in *M. oryzae* with HIK-gene-specific probes. Genomic DNA of *M. oryzae* strain 70-15 and the mutants were isolated and restricted with corresponding restriction enzymes. The probes which we used for hybridization with the genomic DNA of the wild-type strain and the corresponding mutant strains were always identical. “*” marked the position of the hybridization of the probes. **Figure S2.** Protein domain scheme of the sequences which were used for the phylogenetic analysis of the two-component hybrid histidine kinases of fungi. Bf (*Botryotinia* *f**uckeliana*), Ca (*Candida albicans*), Ch (*Cochliobolus heterostrophus*), Ec (*Escherichia coli*), En (*Emericella nidulans*), Gm (*Gibberella moniliformis*), Mg (*Mycosphaerella graminicola*), Mo (*Magnaporthe oryzae*), Nc (*Neurospora crassa*), Sc (*Saccharomyces cerevisiae*). HisKA, histidine kinase domain; REC, regulatory domain; HATPase, histidine-ATPase domain; ATPase, ATPase domain; PKc, protein kinase domain; GAF, GAF domain; HAMP, HAMP domain; PAS, PAS domain; PASF, PASF domain; PAC, PAC domain; PHY, phytochrome domain; TM, transmembrane domain. **Figure S3.** Vegetative growth of the *Magnaporthe oryzae* wild-type strain 70-15 and the HIK mutants on complete medium. The fungal strains were grown on complete medium (CM) with additional stress inducing agents NaCl, sorbitol, NaNO_2_, CoCl_2_, or CuSO_4_ for 10 days at 26°C. **Figure S4.** Vegetative growth of the *Magnaporthe oryzae* wild-type strain 70-15 and the HIK mutants on minimal medium. The fungal strains were grown on minimal medium (MM) with additional stress inducing agents NaCl, sorbitol, NaNO_2_, CoCl_2_, CuSO_4_, or H_2_O_2_ for 10 days at 26°C. **Table S1.** List of oligonucleotides used in this study. **Table S2.** Vegetative growth of the *Magnaporthe oryzae* wild-type strain 70-15 and the HIK mutants. **Table S3.** GeneBank accession numbers or the gene name from the *Magnaporthe* comparative database of the two-component hybrid histidine kinases used for the phylogenetic analysis. **Data S1.** Strategies of Inactivating Genes Within the *Magnaporthe oryzae* genome.
