# Supplementary material for: Histidine kinases mediate differentiation, stress response, and pathogenicity in Magnaporthe oryzae
Source: Microbiologyopen. 2014 Aug 8;3(5):668–87. doi: 10.1002/mbo3.197 (PMC4234259; doi:10.1002/mbo3.197)
Supplement: Supplementary file 1 [file mbo30003-0668-sd1.docx]

**Supporting information**

Article title: Histidine kinases mediate differentiation, stress response and pathogenicity in *Magnaporthe oryzae*

Authors: Stefan Jacob, Andrew J. Foster, Alexander Yemelin and Eckhard Thines

Article acceptance date: Click here to enter a date.

The following Supporting Information is available for this article:

**Fig. S1** Southern blot analysis of gene inactivation mutants in *M. oryzae* with HIK-gene specific probes.

**Fig. S2** Protein domain scheme of the sequences which were used for the phylogenetic analysis of the two-component hybrid histidine kinases of fungi.

**Fig. S3** Vegetative growth of the *Magnaporthe oryzae* wildtype strain 70-15 and the HIK mutants on complete medium. The fungal strains were grown on complete medium (CM) with additional stress inducing agents NaCl, sorbitol, NaNO_2_, CoCl_2_ or CuSO_4_ for 10 days at 26°C.

**Fig. S3** Vegetative growth of the *Magnaporthe oryzae* wildtype strain 70-15 and the HIK mutants on minimal medium. The fungal strains were grown on minimal medium (MM) with additional stress inducing agents NaCl, sorbitol, NaNO_2_, CoCl_2_, CuSO_4_ or H_2_O_2_ for 10 days at 26°C.

**Table S1** List of oligonucleotides used in this study.

**Table S2** Vegetative growth of the *Magnaporthe oryzae* wildtype strain 70-15 and the HIK mutants.

**Table S3** GeneBank accession numbers or the gene name from the *Magnaporthe* comparative Database of the two-component hybrid histidine kinases used for the phylogenetic analysis.

**Methods S1** Strategies of inactivating genes within the *Magnaporthe oryzae* genome.

**Figure S1: Southern blot analysis of gene inactivation mutants in *M. oryzae* with HIK-gene specific probes.** Genomic DNA of *M. oryzae* strain 70-15 and the mutants were isolated and restricted with corresponding restriction enzymes. The probes which we used for hybridization with the genomic DNA of the wildtype strain and the corresponding mutant strains were always identical. “*” marked the position of the hybridization of the probes.
















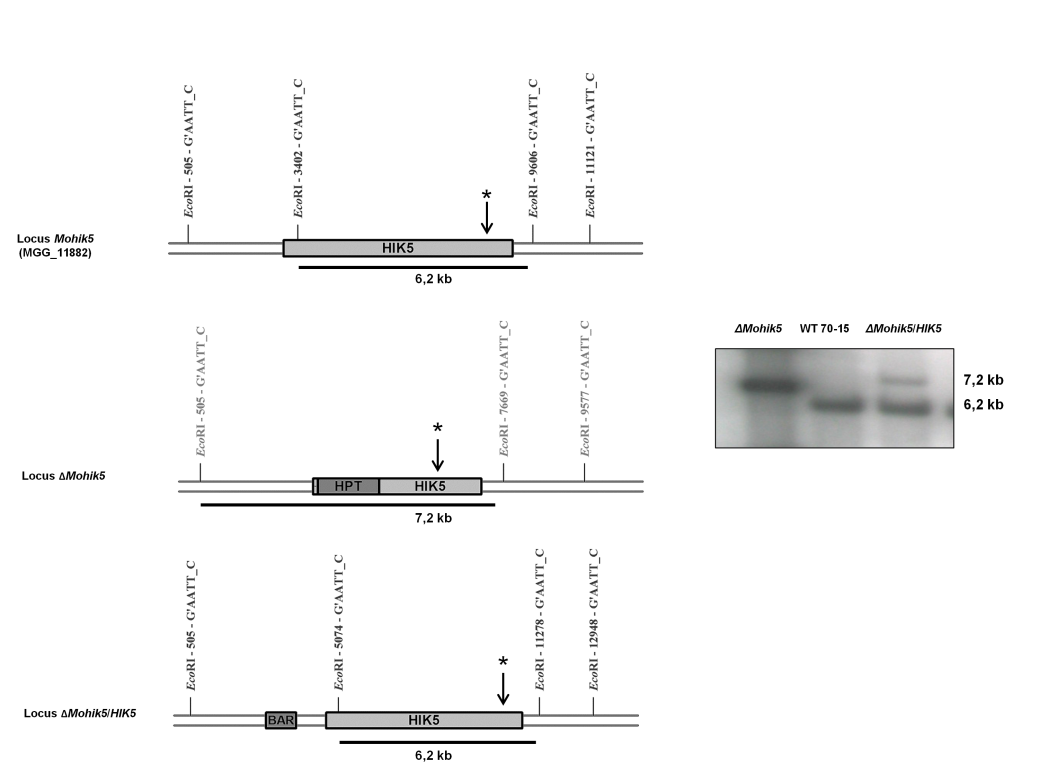

















**Figure S2: Protein domain scheme of the sequences which were used for the phylogenetic analysis of the two-component hybrid histidine kinases of fungi.** Bf (*Botryotinia fuckeliana*), Ca (*Candida albicans*), Ch (*Cochliobolus heterostrophus*), Ec (*Escherichia coli*), En (*Emericella nidulans*), Gm (*Gibberella moniliformis*), Mg (*Mycosphaerella graminicola*), Mo (*Magnaporthe oryzae*), Nc (*Neurospora crassa*), Sc (*Saccharomyces cerevisiae*). HisKA = histidine kinase domain, REC = regulatory domain, HATPase = histidine-ATPase domain, ATPase = ATPase domain, PKc = protein kinase domain, GAF = GAF domain, HAMP = HAMP domain, PAS = PAS domain, PASF = PASF domain, PAC = PAC domain, PHY = phytochrome domain, TM = transmembrane domain.


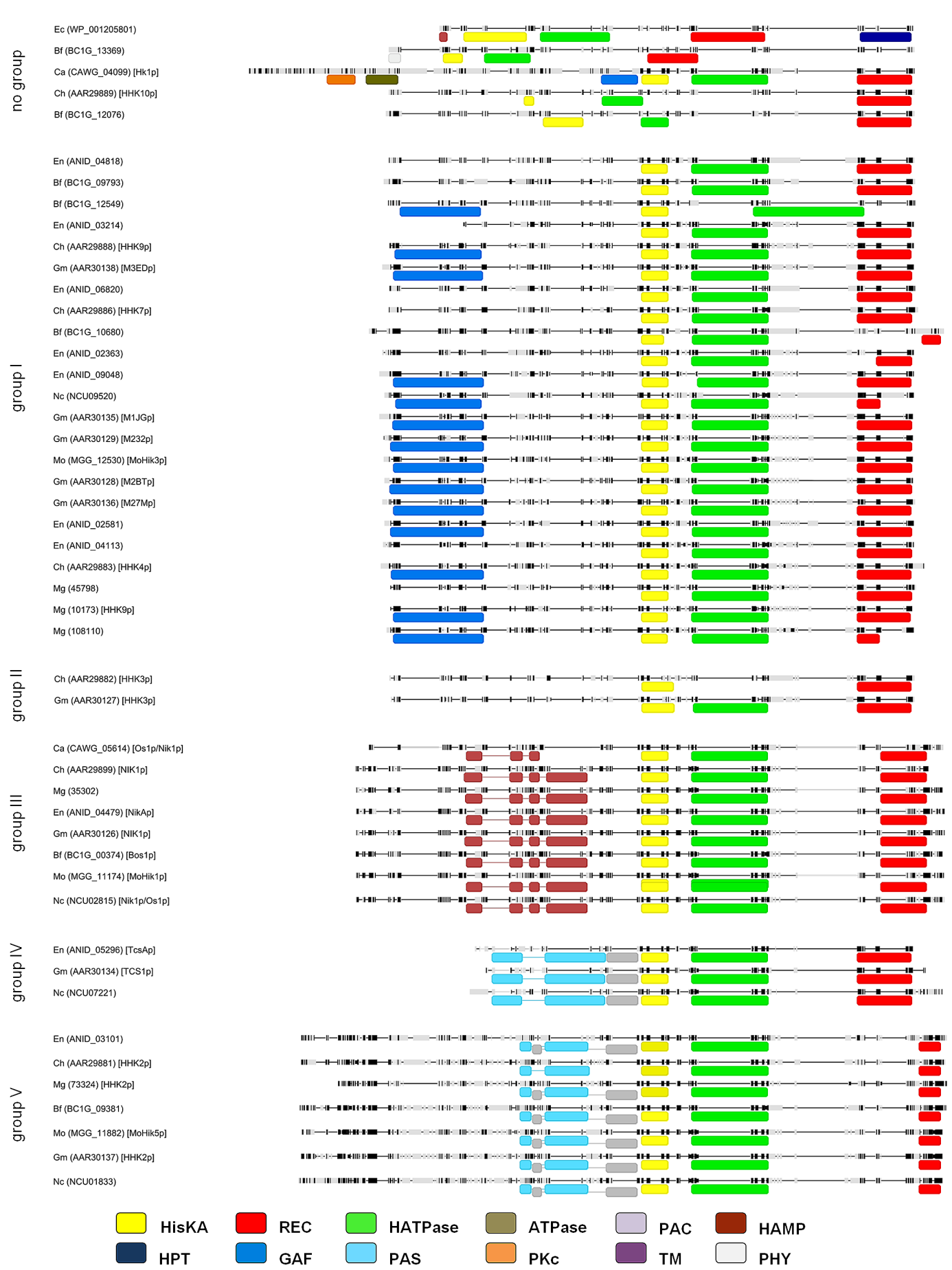


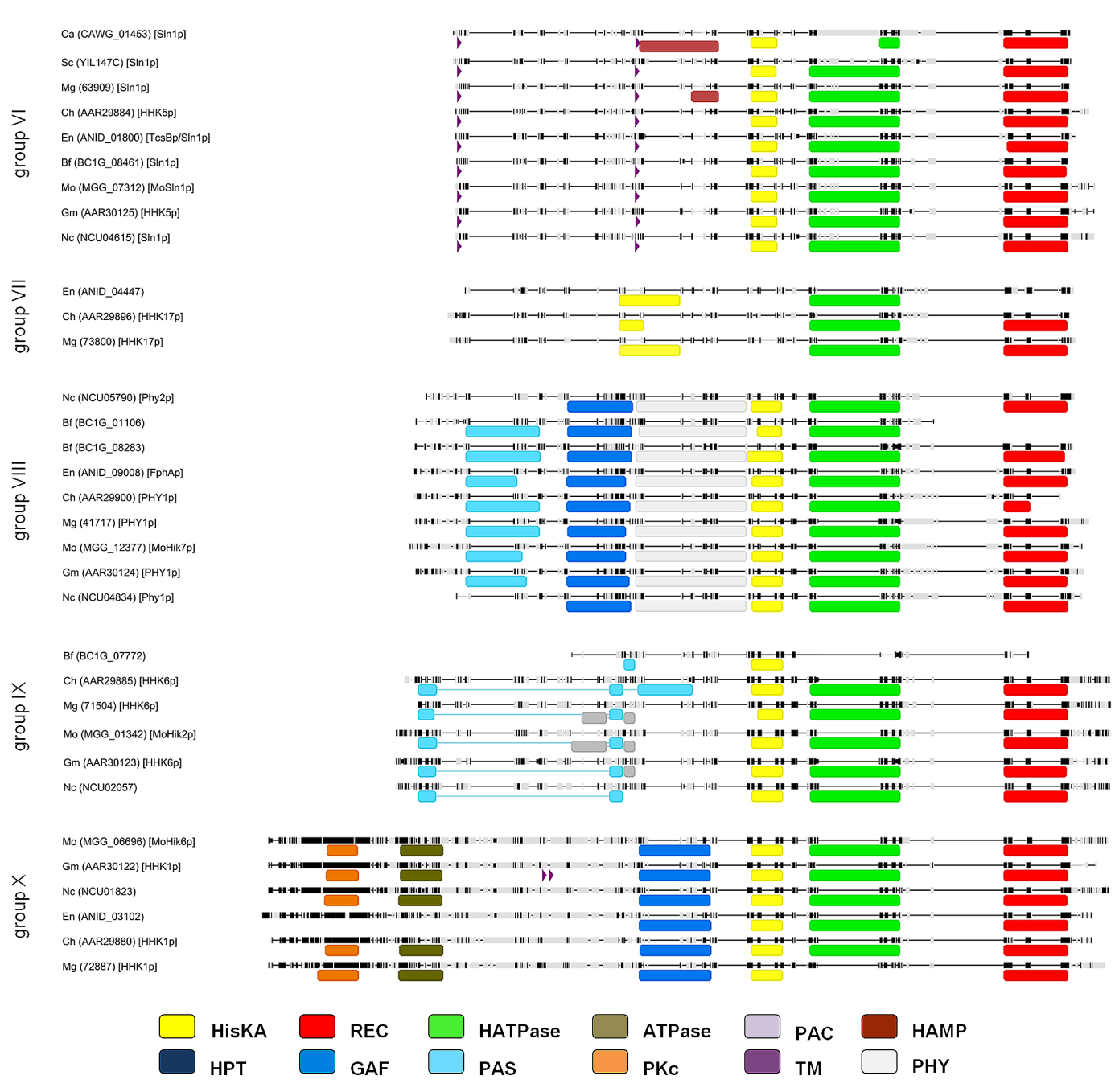


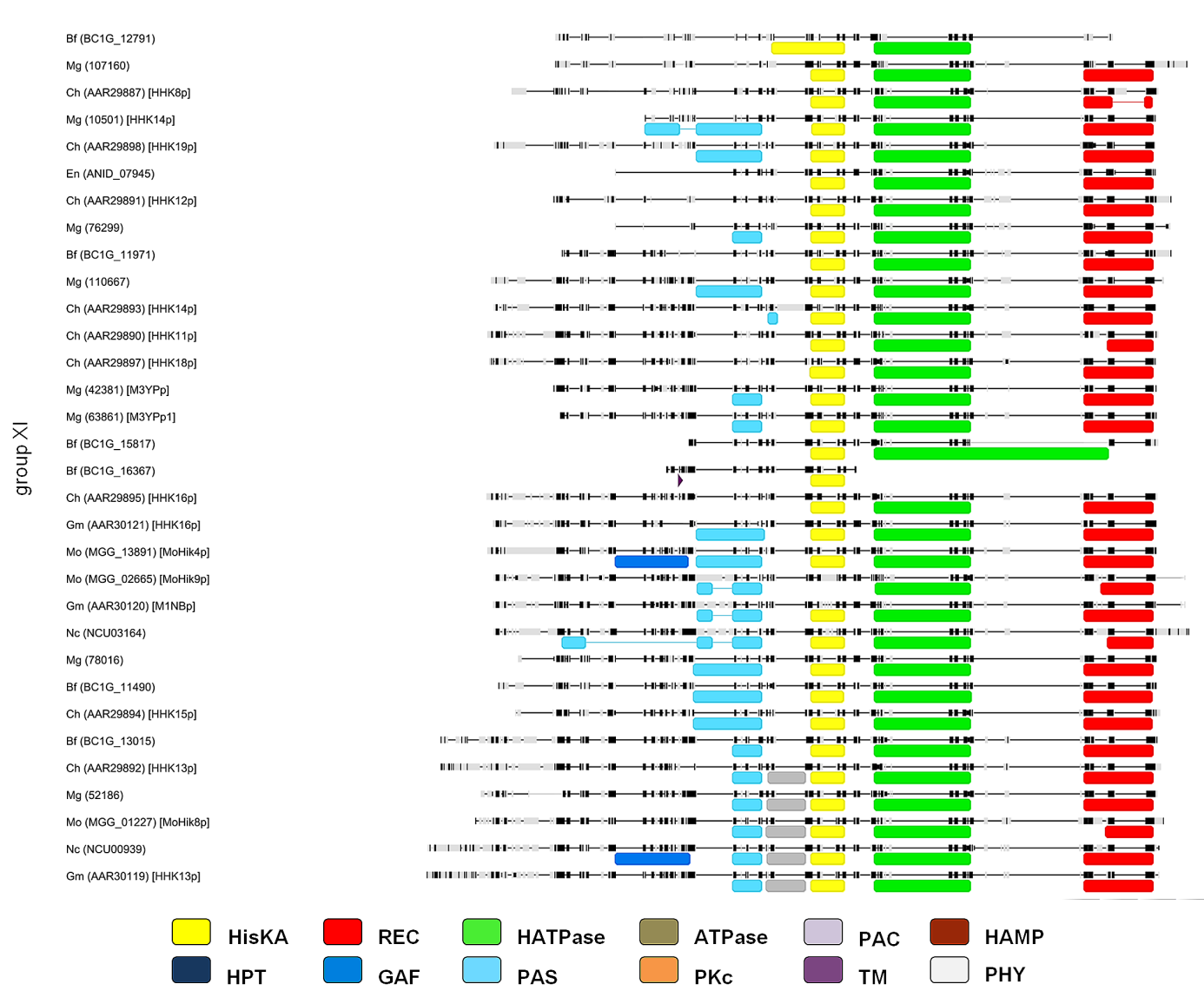


Figure S3: Vegetative growth of the *Magnaporthe oryzae* wildtype strain 70-15 and the HIK mutants on complete medium. The fungal strains were grown on complete medium (CM) with additional stress inducing agents NaCl, sorbitol, NaNO_2_, CoCl_2_ or CuSO_4_ for 10 days at 26°C.

**
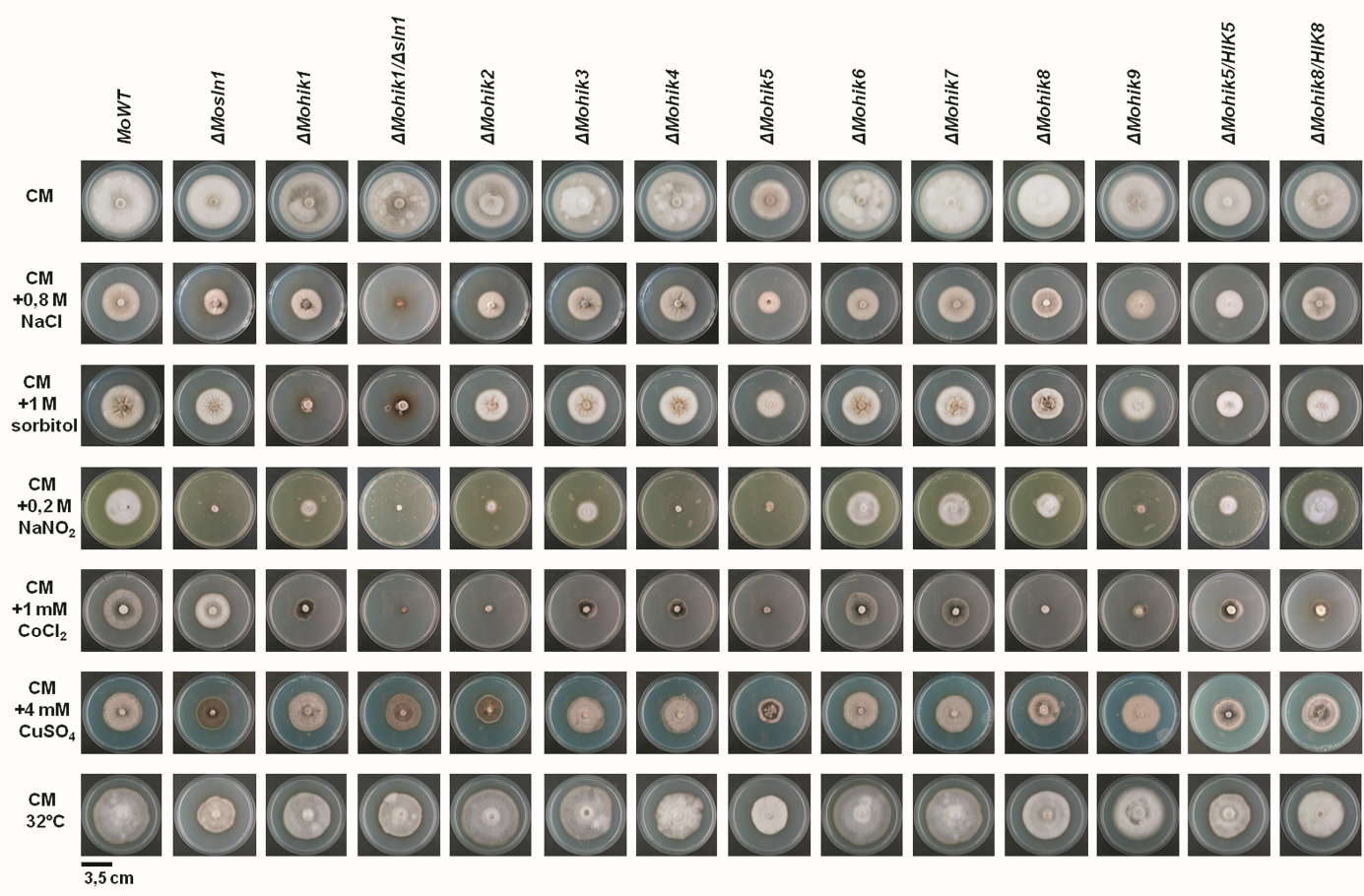
**

**Figure S4: Vegetative growth of the *Magnaporthe oryzae* wildtype strain 70-15 and the HIK mutants on minimal medium.** The fungal strains were grown on minimal medium (MM) with additional stress inducing agents NaCl, sorbitol, NaNO_2_, CoCl_2_, CuSO_4_ or H_2_O_2_ for 10 days at 26°C.


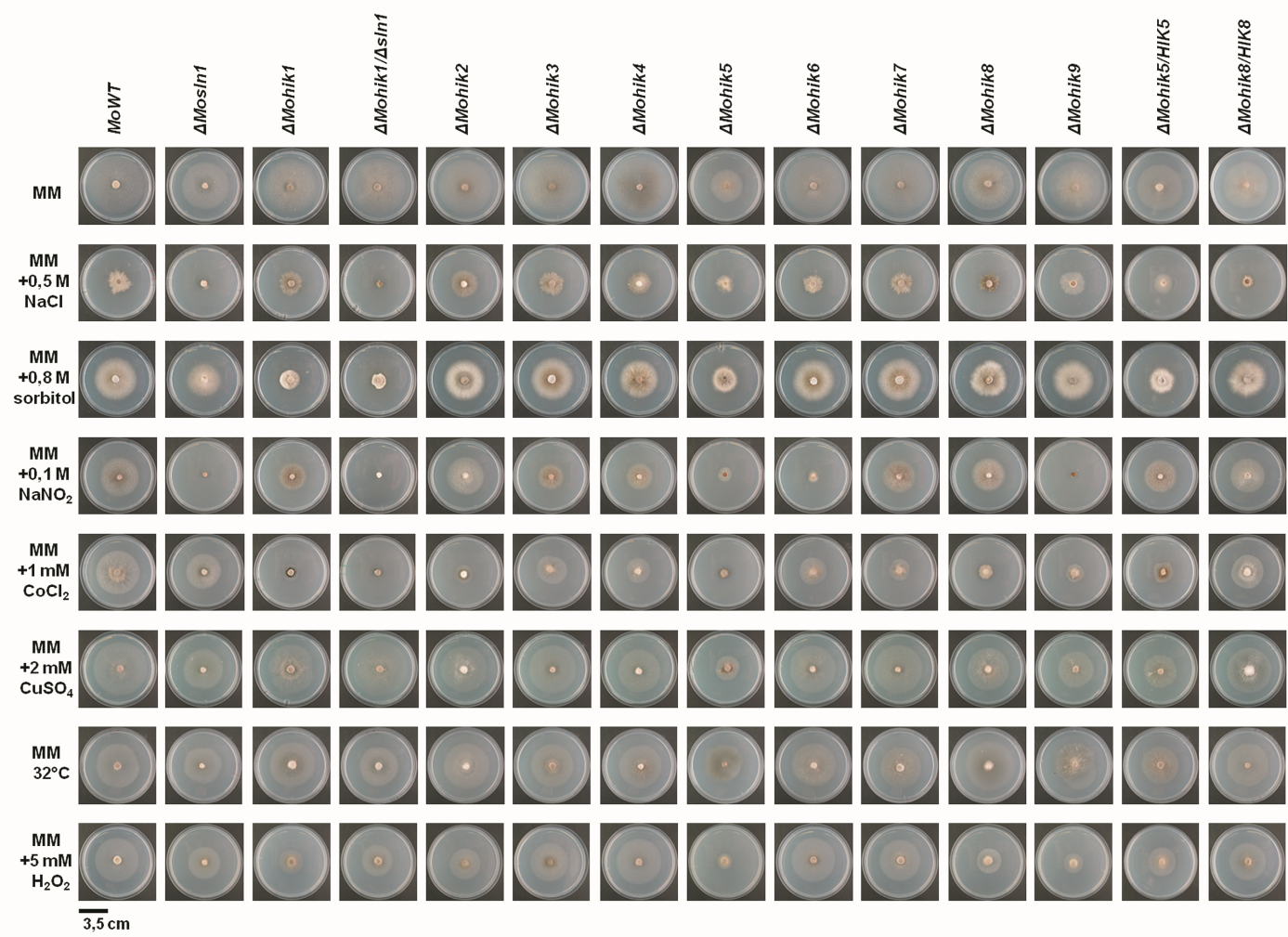


**Table S1: List of oligonucleotides used in this study.**

| **name** | **sequence (5‘→ 3‘)** |
| --- | --- |
| SLN1-PCR-for | GGCCGCTGCGCTACTGTGAG |
| SLN1-PCR-rev | GCCGAGCCTATGATGAGATGAAGAG |
| SLN1-DIG-for | GTTATCCGGCCCGACTACAG |
| SLN1-DIG-rev | GCTTGCCGCCCTGGTGATAA |
| HIK1-PCR-for | CGGGCTGTGTCGATTGAGTC |
| HIK1-PCR-rev | AAGATTGAGGCTAAGCGGATGGTT |
| HIK1-DIG-for | GGTTGACGAGCCTCTTGGAAATAG |
| HIK1-DIG-rev | GGATGGCCAGAAGGTCGTGTT |
| HIK2-PCR-for | GGCCGCCATGATTTCTACAACGAC |
| HIK2-PCR-rev | GCGCCTCCAAGCTGTATGATG |
| HIK2-DIG-for | CCCAGCGCAAAGCCCCCTCTA |
| HIK2-DIG-rev | CCGTGGTCTTTTGGCCTTTTGTC |
| HIK3-PCR-for | GCCGCTTGCTTGCTTGCTTTAC |
| HIK3-PCR-rev | GGTAAAATCGCAAACCTCTG |
| HIK3-DIG-for | GGTTGTCGTCGACCAGTAG |
| HIK3-DIG-rev | CGACCTCCTGCCAAAGAAAATAAGCC |
| HIK4-PCR-for | GCCAGCAAACTCCGCCCAGATATG |
| HIK4-PCR-rev | CCGTATTTATCCTCCCTGCG |
| HIK4-DIG-for | GGATTTTGCCCCTTGTAGACAG |
| HIK4-DIG-rev | GTCTATTCCGTGAAAAACAAC |
| HIK5-PCR-for | GATTACAGCTGCTCAGAATTC |
| HIK5-PCR-rev | GAGTCGCTAAGACCATCATCG |
| HIK5-DIG-for | GTTGACATGTCGGGCATTTC |
| HIK5-DIG-rev | GCCGAGCCTATGATGAGATGAAGAG |
| HIK6-PCR-for | CAAGAACTTAGGCACATGCAAG |
| HIK6-PCR-rev | GAATCGGATTTGATCAGCAAAC |
| HIK6-DIG-for | CATCTGGGATGAGGACAGTC |
| HIK6-DIG-rev | CGACAGGCTCTTCGATTAG |
| HIK7-PCR-for | CCTTTCATCTCGACAAAAATC |
| HIK7-PCR-rev | CATCGACACATTCTTGTGTAG |
| HIK7-DIG-for | GTTTACACAAACCAGTCAACCG |
| HIK7-DIG-rev | GTTGTTAACCAAGGACCGC |
| HIK8-PCR-for | CAGCTTTTCACTCCGTTCAC |
| HIK8-PCR-rev | CCCGTCACACTTTTTTAATAGC |
| HIK8-DIG-for | GGAAGCAGTCAGCTACACTCC |
| HIK8-DIG-rev | CCTTCAAAGGGCCAACTTG |
| HIK8-comp-for | CTATGCCATCGCGATCGTC |
| HIK9-PCR-for | GTACGTTACCCACAGTAATTCTGAG |
| HIK9-PCR-rev | GTTACCTGTAGCAAACCCG |
| HIK9-DIG-for | CCCAGTCAGTCAGTTTGTTTC |
| HIK9-DIG-for | GATTCAATCCCCCAAATTC |
| HOG1-PCR-for | CTCGCCCCAATTCTCATCGTCTTAACGA |
| HOG1-PCR-rev | CAGTAGAGTAAGCCAGCCGCCTAGCTCTAGC |
| HOG1-DIG-for | CACGATACCACGATTACACC |
| HOG1-DIG-rev | GACCTCATACTCTGCATCTC |

Table S2: Vegetative growth of the *Magnaporthe oryzae* wildtype strain 70-15 and the HIK mutants. The vegetative growth assays were conducted on complete medium (CM) and on minimal medium (MM), and on CM and MM inclusive NaCl, sorbitol, NaNO_2_, CoCl_2_, CuSO_4_, H_2_O_2_ for 10 days at 26°C. In addition equivalent experiments were set up on CM and MM for 10 days at 32°C. The table shows the colony diameter [mm]. The standard deviation was calculated out of three experiments with five replicates each.

|  |  | *MoWT* | *ΔMosln1* | *ΔMohik1* | *ΔMohik1/Δsln1* | *ΔMohik2* | *ΔMohik3* | *ΔMohik4* | *ΔMohik5* | *ΔMohik6* | *ΔMohik7* | *ΔMohik8* | *ΔMohik9* | *ΔMohik5/HIK5* | *ΔMohik8/HIK8* |
| --- | --- | --- | --- | --- | --- | --- | --- | --- | --- | --- | --- | --- | --- | --- | --- |
| Koloniedurchmesser [mm] | CM | 79±3 | 65±2 | 72±2 | 76±2 | 69±2 | 74±3 | 72±2 | 49±1 | 75±3 | 76±2 | 62±2 | 72±3 | 54±2 | 69±2 |
|  | CM+0,8 M NaCl | 47±2 | 29±0 | 38±2 | 11±1 | 34±3 | 43±2 | 44±2 | 25±2 | 40±3 | 46±2 | 37±2 | 36±2 | 37±2 | 42±2 |
|  | CM+1 M sorbitol | 53±2 | 44±2 | 16±1 | 14±2 | 40±2 | 47±2 | 47±3 | 35±2 | 48±1 | 48±2 | 38±3 | 42±2 | 36±1 | 41±2 |
|  | CM+0,2 M NaNO_2_ | 46±2 | 11±1 | 25±2 | 11±2 | 20±2 | 30±2 | 0 | 12±2 | 44±3 | 42±2 | 30±2 | 15±1 | 25±2 | 42±3 |
|  | CM+1 mM CoCl_2_ | 45±2 | 42±3 | 27±2 | 48±2 | 0 | 25±2 | 25±1 | 10±2 | 40±2 | 33±3 | 10±2 | 17±1 | 25±2 | 21±3 |
|  | CM+4 mM CuSO_4_ | 48±3 | 40±2 | 46±2 | 43±2 | 33±3 | 43±2 | 45±2 | 30±3 | 43±2 | 44±3 | 38±2 | 46±2 | 38±2 | 41±2 |
|  | CM 32°C | 65±2 | 45±2 | 51±2 | 55±2 | 64±4 | 69±3 | 55±2 | 44±2 | 71±2 | 68±2 | 57±2 | 60±3 | 52±3 | 55±2 |
|  |  | | | | | | | | | | | | | | |
|  | MM | 72±2 | 61±2 | 70±2 | 71±2 | 66±2 | 69±4 | 74±2 | 45±2 | 70±3 | 72±2 | 63±3 | 67±2 | 61±3 | 62±2 |
|  | MM+0,5 M NaCl | 31±2 | 0 | 30±1 | 0 | 35±2 | 29±1 | 28±2 | 24±2 | 26±1 | 30±2 | 22±2 | 29±1 | 24±2 | 26±1 |
|  | MM+0,8 M sorbitol | 56±3 | 49±2 | 25±2 | 19±1 | 51±2 | 52±2 | 48±2 | 35±1 | 55±2 | 54±2 | 45±2 | 51±2 | 35±2 | 51±2 |
|  | MM+0,1 M NaNO_2_ | 45±3 | 0 | 35±2 | 0 | 48±3 | 35±2 | 35±2 | 18±1 | 15±2 | 44±2 | 40±2 | 0 | 41±3 | 39±2 |
|  | MM+1 mM CoCl_2_ | 40±2 | 30±2 | 22±2 | 0 | 20±1 | 36±2 | 27±2 | 12±2 | 31±3 | 26±2 | 11±1 | 20±1 | 20±1 | 37±2 |
|  | MM+2 mM CuSO_4_ | 67±2 | 46±1 | 59±2 | 72±2 | 56±2 | 59±3 | 50±1 | 28±2 | 54±2 | 59±1 | 51±2 | 42±2 | 40±2 | 56±2 |
|  | MM 32°C | 54±1 | 45±2 | 55±2 | 50±2 | 55±2 | 53±3 | 55±2 | 44±1 | 53±2 | 53±2 | 62±2 | 51±2 | 44±2 | 49±2 |
|  | MM+5 mM H_2_O_2_ | 55±1 | 41±2 | 30±1 | 39±2 | 39±2 | 48±1 | 56±2 | 18±1 | 55±2 | 50±1 | 29±2 | 23±2 | 39±2 | 43±2 |

Table S3: GeneBank accession numbers or the gene name from the *Magnaporthe* comparative Database of the two-component hybrid histidine kinases used for the phylogenetic analysis.

| *B.fuckeliana* | *C.albicans* | *C.heterostrophus* | *E.nidulans* | *G.moniliformis* | *M.oryzae* | *M.graminicola* | *N.crassa* | *S.cerevisiae* |
| --- | --- | --- | --- | --- | --- | --- | --- | --- |
| BC1G_11971 | CAWG_05614  (Os1p/Nik1p) | AAR29880  (HHK1p) | ANID_04479  (NikAp) | AAR30119  (HHK13p) | MGG_07312  (MoSln1p) | 73324  (HHK2p) | NCU02815  (Nik1p/Os1p) | YIL147C  (Sln1p) |
| BC1G_13015 | CAWG_04099  (Hk1p) | AAR29881  (HHK2p) | ANID_05296  (TcsAp) | AAR30120  (M1NBp) | MGG_11174  (MoHik1p) | 72887  (HHK1p) | NCU01823 |  |
| BC1G_12076 | CAWG_01453  (Sln1p) | AAR29882  (HHK3p) | ANID_01800  (TcsBp/Sln1p) | AAR30121  (HHK16p) | MGG_01342  (MoHik2p) | 35302 | NCU01833 |  |
| BC1G_01106 |  | AAR29883  (HHK4p) | ANID_02581 | AAR30122  (HHK1p) | MGG_12530  (MoHik3p) | 41717 | NCU00939 |  |
| BC1G_13369 |  | AAR29884  (HHK5p) | ANID_02363 | AAR30123  (HHK6p) | MGG_13891  (MoHik4p) | 52186 | NCU02057 |  |
| BC1G_08283 |  | AAR29885  (HHK6p) | ANID_03101 | AAR30124  (PHY1p) | MGG_11882  (MoHik5p) | 63909  (Sln1p) | NCU03164 |  |
| BC1G_00374  (Bos1p) |  | AAR29886  (HHK7p) | ANID_03102 | AAR30125  (HHK5p) | MGG_06696  (MoHik6p) | 71504  (HHK6p) | NCU04615  (Sln1p) |  |
| BC1G_09381 |  | AAR29887  (HHK8p) | ANID_03214 | AAR30126  (NIK1p) | MGG_12377  (MoHik7p) | 52186 | NCU04834  (Phy1p) |  |
| BC1G_11490 |  | AAR29888  (HHK9p) | ANID_04113 | AAR30127  (HHK3p) | MGG_01227  (MoHik8p) | 45798 | NCU05790  (Phy2p) |  |
| BC1G_12549 |  | AAR29889  (HHK10p) | ANID_04447 | AAR30128  (M2BTp) | MGG_02665  (MoHik9p) | 10501  (HHK14p) | NCU07221 |  |
| BC1G_08461  (Sln1p) |  | AAR29890  (HHK11p) | ANID_04818 | AAR30129  (M232p) |  | 42381  (M3YPp) | NCU09520 |  |
| BC1G_15817 |  | AAR29891  (HHK12p) | ANID_06820 | AAR30134  (TCS1p) |  | 63861  (M3YPp) |  |  |
| BC1G_12791 |  | AAR29892  (HHK13p) | ANID_07945 | AAR30135  (M1JGp) |  | 78016 |  |  |
| BC1G_10680 |  | AAR29893  (HHK14p) | ANID_09048 | AAR30136  (M27Mp) |  | 73800  (HHK17p) |  |  |
| BC1G_07772 |  | AAR29894  (HHK15p) | ANID_09008  (FphAp) | AAR30137  (HHK2p) |  | 76299 |  |  |
| BC1G_09793 |  | AAR29895  (HHK16p) |  | AAR30138  (M3EDp) |  | 110667 |  |  |
| BC1G_16367 |  | AAR29896  (HHK17p) |  |  |  | 107160 |  |  |
|  |  | AAR29897  (HHK18p) |  |  |  | 10173  (HHK9p) |  |  |
|  |  | AAR29898  (HHK19p) |  |  |  | 108110 |  |  |
|  |  | AAR29899  (NIK1p) |  |  |  |  |  |  |
|  |  | AAR29900  (PHY1p) |  |  |  |  |  |  |

As „outgroup“ the sequence of *E.coli* (WP_00120580) was used.

Databases:

- *B. fuckeliana*: Broad Institut (http://www.broadinstitute.org/scientific-community/data)
- *C. albicans*: Broad Institut (http://www.broadinstitute.org/scientific-community/data)
- *C. heterostrophus*: NCBI Protein-ID (http://www.ncbi.nlm.nih.gov/genbank/)
- *E. coli:* NCBI Protein-ID (http://www.ncbi.nlm.nih.gov/genbank/)
- *E. nidulans*: Broad Institut (http://www.broadinstitute.org/scientific-community/data)
- *G. moniliformis*: NCBI Protein-ID (http://www.ncbi.nlm.nih.gov/genbank/)
- *M. graminicola*: JGI protein ID (http://genome.jgi-psf.org/pages/search-for-genes.jsf?organism=Mycgr3)
- *M. oryzae*: Broad Institut (http://www.broadinstitute.org/scientific-community/data)
- *N. crassa*: Broad Institut (http://www.broadinstitute.org/scientific-community/data)
- *S. cerevisiae*: SGD (http://www.yeastgenome.org/)

**Methods S1: Strategies of inactivating genes within the *Magnaporthe oryzae* genome.**

In case of *ΔMosln1* a 4651 bp PCR product was amplified by using the primers *SLN1*-PCR-for and *SLN1*-PCR-rev from genomic DNA of *M. oryzae* 70-15 and cloned into *pGEMTeasy* (Promega, Mannheim; Germany) giving the vector *pGEMT+SLN1*. *pGEMT+SLN1* was restricted with *Hind*III/*Sma*I and a 2857 bp fragment of the coding sequence was replaced by a *Hind*III/*Sma*I restricted *BAR* cassette from *pCAMB+BAR* (Kramer *et al*., 2009) to give *pGEMT+SLN1+BAR*. The *Not*I/*Spe*I restricted fragment of *pGEMT+SLN1+BAR* was cloned into *Psp*OMI/*Spe*I restricted *pCAMBIA0380* to give the gene inactivation vector *pCAMB+SLN1+BAR*.

In case of *ΔMohik1* a 3320 bp PCR product was amplified with the primers *HIK1*-PCR-for and *HIK1*-PCR-rev from genomic DNA of *M. oryzae* 70-15 and cloned into *pGEMTeasy* giving the vector *pGEMT+HIK1*. *pGEMT+HIK1* was restricted with *Sma*I and a *Hpa*I restricted *HPT* cassette from pCAMB+*HPT* (Odenbach *et al*., 2007) was inserted to give *pGEMT+HIK1+HPT*. The *Not*I restricted fragment of *pGEMT+HIK1+HPT* was cloned into *Psp*OMI restricted *pCAMBIA0380* to give the gene disruption vector *pCAMB+HIK1+HPT*.

In case of *ΔMohik1/Δsln1* the gene inactivation vector *pCAMB+SLN1+BAR* was used to transform the gene inactivation mutant *ΔMohik1*.

In case of *ΔMohik2* a 3133 bp PCR product was amplified with the primers *HIK2*-PCR-for and *HIK2*-PCR-rev from genomic DNA of *M. oryzae* 70-15 and cloned into *pGEMTeasy* giving the vector *pGEMT+HIK2*. *pGEMT+HIK2* was restricted with *Hpa*I and a *Hpa*I restricted *HPT* cassette from *pCAMB+HPT* was inserted to give *pGEMT+HIK2+HPT*. The *Not*I restricted fragment of *pGEMT+HIK2+HPT* was cloned into *Psp*OMI restricted *pCAMBIA0380* to give the gene disruption vector *pCAMB+HIK2+HPT*.

In case of *ΔMohik3* a 3849 bp PCR product was amplified with the primers *HIK3*-PCR-for and *HIK3*-PCR-rev from genomic DNA of *M. oryzae* 70-15 and cloned into *pGEMTeasy* giving the vector *pGEMT+HIK3*. *pGEMT+HIK3* was restricted with *Avr*II/*Bgl*II and a 3631 bp fragment of the sequence was replaced by a *Avr*II/*Bgl*II restricted *HPT* cassette from *pCAMB+HPT+Hind* (Kramer *et al*., 2009) to give *pGEMT+HIK3+HPT*. The *Not*I/*Spe*I restricted fragment of *pGEMT+HIK3+HPT* was cloned into *Psp*OMI/*Spe*I restricted *pCAMBIA0380* to give the gene inactivation vector *pCAMB+HIK3+HPT*.

In case of *ΔMohik4* a 4196 bp PCR product was amplified with the primers *HIK4*-PCR-for and *HIK4*-PCR-rev from genomic DNA of *M. oryzae* 70-15 and cloned into *pGEMTeasy* giving the vector *pGEMT+HIK4*. *pGEMT+HIK4* was restricted with *Xba*I/*Xho*I and a 3485 bp fragment of the sequence was replaced by a *XbaI/XhoI* restricted *HPT* cassette *from pCB1636* (Sweigard *et al*., 1997) to *give pGEMT+HIK4+HPT*. The *Not*I/*Avr*II restricted fragment of *pGEMT+HIK4+HPT* was cloned into *Psp*OMI/*Avr*II restricted *pCAMBIA0380* to give the gene inactivation vector *pCAMB+HIK4+HPT*.

In case of *ΔMohik5* a 7222 bp PCR product was amplified with the primers *HIK5*-PCR-for and *HIK5*-PCR-rev from genomic DNA of *M. oryzae* 70-15 and cloned into *pGEMTeasy* giving the vector *pGEMT+HIK5*. *pGEMT+HIK5* was restricted with *SgrA*I/*Bgl*II and a 4000 bp fragment of the sequence was replaced by a *Xma*I/*Bgl*II restricted *HPT* cassette from pCAMB+HPT+Hind to give *pGEMT+HIK5+HPT*. The *Not*I/*Spe*I restricted fragment of *pGEMT+HIK5+HPT* was cloned into *Psp*OMI/*Spe*I restricted *pCAMBIA0380* to give the gene inactivation *vector pCAMB+HIK5+HPT*.

In case of *ΔMohik5/HIK5* a 7220 bp PCR product was amplified with the primers *HIK5*-PCR-for and *HIK5*-PCR-rev from genomic DNA of *M. oryzae* 70-15 and cloned into *pJET1.2/blunt* giving the vector *pJET+HIK5(comp)*. *pJET+HIK5(comp)* was restricted with *Not*I/*Swa*I and a 7554 bp fragment of the promoter and coding region was cloned into a *Psp*OMI/*Sma*I restricted *pCAMB+BAR* to give *pCAMB+HIK5*(*komp*)+*BAR*. *pCAMB+HIK5*(*komp*)+*BAR* was used to transform the gene inactivation mutant *ΔMohik5*.

In case of *ΔMohik6* a 9486 bp PCR product was amplified with the primers *HIK6*-PCR-for and *HIK6*-PCR-rev from genomic DNA of *M. oryzae* 70-15 and cloned into *pGEMTeasy* giving the vector *pGEMT-HIK6*. *pGEMT+HIK6* was restricted with *Nhe*I/*Pml*I and a 6491 bp fragment of the sequence was replaced by a *Nhe*I/*Sma*I restricted *HPT* cassette from *pCAMB+HPT+Hind* to give *pGEMT+HIK6+HPT*. The *Not*I/*Afe*I restricted fragment of *pGEMT-HIK6-HPT* was cloned into *Psp*OMI/*Sma*I restricted *pCAMBIA0380* to give the gene inactivation vector *pCAMB+HIK6+HPT*.

In case of *ΔMohik7* a 6325 bp PCR product was amplified with the primers *HIK7*-PCR-for and *HIK7*-PCR-rev from genomic DNA of *M. oryzae* 70-15 and cloned into *pGEMTeasy* giving the vector *pGEMT+HIK7*. *pGEMT+HIK7* was restricted with *Bgl*II/*Bam*HI and a 3523 bp fragment of the sequence was replaced by a *Bgl*II/*Bam*HI restricted *HPT* cassette from *pCAMB+HPT+Hind* to give *pGEMT+HIK7+HPT*. The *Not*I/*Xba*I restricted fragment of *pGEMT-HIK7-HPT* was cloned into *Psp*OMI/*Avr*II restricted *pCAMBIA0380* to give the gene inactivation vector *pCAMB+HIK7+HPT*.

In case of *ΔMohik8* a 5984 bp PCR product was amplified with the primers *HIK8*-PCR-for and *HIK8*-PCR-rev from genomic DNA of *M. oryzae* 70-15 and cloned into *pGEMTeasy* giving the vector *pGEMT+HIK8*. *pGEMT+HIK8* was restricted with *Bgl*II/*Hpa*I and a 3630 bp fragment of the sequence was replaced by a *Bam*HI/*Pml*I restricted *HPT* cassette from *pCAMB+HPT+Hind* to give *pGEMT+HIK8+HPT*. The *Eco*RV/*Xba*I restricted fragment *of pGEMT+HIK8+HPT* was cloned into *Sma*I/*Spe*I restricted *pCAMBIA0380* to give the gene inactivation vector *pCAMB+HIK8+HPT*.

In case of *ΔMohik8/HIK8* a 6824 bp PCR product was amplified with the primers *HIK8*-comp-for and *HIK8*-PCR-rev from genomic DNA of *M. oryzae* 70-15 and cloned into *pJET1.2/blunt* giving the vector *pJET+HIK8(comp)*. *pJET+HIK8(comp)* was restricted with *Pvu*I/*Spe*I and a 6498 bp fragment of the promoter and coding region was cloned into a *Pvu*I/*Spe*I restricted *pCAMB+BAR* to give *pCAMB+HIK8*(*komp*)+*BAR*. *pCAMB+HIK8*(*komp*)+*BAR* was used to transform the gene inactivation mutant *ΔMohik8*.

In case of *ΔMohik9* a 5919 bp PCR product was amplified with the primers *HIK9*-PCR-for and *HIK9*-PCR-rev from genomic DNA of *M. oryzae* 70-15 and cloned into *pGEMTeasy* giving the vector *pGEMT+HIK9*. *pGEMT+HIK9* was restricted with *Bgl*II/*Xba*I and a 4115 bp fragment of the sequence was replaced by a *Bgl*II/*Avr*II restricted *HPT* cassette *from pCAMB+HPT+Hind* to give *pGEMT+HIK9+HPT*. The *Not*I/*Spe*I restricted fragment of *pGEMT+HIK9+HPT* was cloned into *Psp*OMI/*Spe*I restricted *pCAMBIA0380* to give the gene inactivation vector *pCAMB+HIK9+HPT*.

In case of *ΔMohog1* a 3047 bp PCR product was amplified with the primers *HOG1*-PCR-for and *HOG1*-PCR-rev from genomic DNA of *M. oryzae* 70-15 and cloned into *pGEMTeasy* giving the vector *pGEMT+HOG1*. *pGEMT+HOG1* was restricted with *Bgl*II/*Avr*II and a 1516 bp fragment of the coding sequence was replaced by a *Bam*HI/*Spe*I restricted *HPT* cassette from *pCAMB+HPT+Sal* (Kramer *et al*., 2009) to give *pGEMT+HOG1+HPT*. The *Not*I/*Spe*I restricted fragment of *pGEMT+HOG1+HPT* was cloned into *Psp*OMI/*Spe*I restricted *pCAMBIA0380* to give the gene inactivation vector *pCAMB+HOG1+HPT*.
